# Insights into the prognosis of lipidomic dysregulation for death risk in patients with coronary artery disease

**DOI:** 10.1002/ctm2.189

**Published:** 2020-09-28

**Authors:** Min Qin, Qian Zhu, Weihua Lai, Qilin Ma, Chen Liu, Xiaoping Chen, Yuelin Zhang, Zixian Wang, Hui Chen, Hong Yan, Heping Lei, Shuyao Zhang, Xuekui Dong, Hong Wang, Min Huang, Qizhou Lian, Shilong Zhong

**Affiliations:** ^1^ Department of Pharmacy Guangdong Provincial People's Hospital Guangdong Academy of Medical Sciences Guangzhou Guangdong P. R. China; ^2^ Guangdong Provincial Key Laboratory of Coronary Heart Disease Prevention Guangdong Provincial People's Hospital Guangdong Academy of Medical Sciences Guangdong Cardiovascular Institute Guangzhou Guangdong P. R. China; ^3^ School of Medicine South China University of Technology Guangzhou Guangdong P. R. China; ^4^ Department of Clinical Pharmacology Xiangya Hospital Central South University Changsha Hunan P. R. China; ^5^ Department of Cardiology The First Affiliated Hospital Sun Yat‐sen University Guangzhou Guangdong P. R. China; ^6^ Department of Emergency Medicine Department of Emergency and Critical Care Medicine Guangdong Provincial People's Hospital Guangdong Academy of Medical Sciences Guangzhou Guangdong P. R. China; ^7^ School of Biology and Biological Engineering South China University of Technology Guangzhou Guangdong P. R. China; ^8^ Guangzhou Red Cross Hospital affiliated to Ji‐Nan University Medical College Guangzhou Guangdong P. R. China; ^9^ Wuhan Metware Biotechnology Co., Ltd. Wuhan Hubei P. R. China; ^10^ School of Pharmaceutical Sciences Institute of Clinical Pharmacology Sun Yat‐Sen University Guangzhou Guangdong P. R. China; ^11^ Department of Medicine The University of Hong Kong Pokfulam Hong Kong

**Keywords:** coronary artery disease, death, left ventricular remodeling, long‐chain polyunsaturated fatty acids, risk stratification, widely targeted lipidomic profiling

## Abstract

**Background:**

Dyslipidaemia contributes to the progression of coronary artery disease (CAD) toward adverse outcomes. Plasma lipidomic measure may improve the prognostic performances of clinical endpoints of CAD. Our research is designed to identify the correlations between plasma lipid species and the risks of death, major adverse cardiovascular event (MACE) and left ventricular (LV) remodeling in patients with CAD.

**Methods:**

A total of 1569 Chinese patients with CAD, 1011 single‐centre patients as internal training cohort, and 558 multicentre patients as external validation cohort, were enrolled. The concentration of plasma lipids in both cohorts was determined through widely targeted lipidomic profiling. Least absolute shrinkage and selection operator Cox and multivariate Cox regressions were used to develop prognostic models for death and MACE, respectively.

**Results:**

Ten (Cer(d18:1/20:1), Cer(d18:1/24:1), PE(30:2), PE(32:0), PE(32:2), PC(O‐38:2), PC(O‐36:4), PC(16:1/22:2), LPC(18:2/0:0) and LPE(0:0/24:6)) and two (Cer(d18:1/20:1) and LPC(20:0/0:0)) lipid species were independently related to death and MACE, respectively. Cer(d18:1/20:1) and Cer(d18:1/24:1) were correlated with LV remodeling (*P* < .05). The lipidic panel incorporating 10 lipid species and two traditional biomarkers for predicting 5‐year death risk represented a remarkable higher discrimination than traditional model with increased area under the curve from 76.56 to 83.65%, continuous NRI of 0.634 and IDI of 0.131. Furthermore, the panel was successfully used in differentiating multicentre patients with low, middle, or high risks (*P* < .0001). Further analysis indicated that the number of double bonds of phosphatidyl choline and the content of carbon atoms of phosphatidyl ethanolamines were negatively associated with death risk.

**Conclusions:**

Improvement in the prediction of death confirms the effectiveness of plasma lipids as predictors to risk classification in patients with CAD. The association between the structural characteristics of long‐chain polyunsaturated fatty acids and death risk highlights the need for mechanistic research that characterizes the role of individual lipid species in disease pathogenesis.

## Graphical abstract

XXXXX.

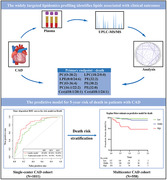

## 1 BACKGROUND

Coronary artery disease (CAD) imposes a major burden on modern society with annual morbidity and mortality comparable to those of cancer.^1^, ^2^, ^3^ Despite the remarkable amelioration of pharmaceutical and operative treatments, estimating and managing the prognostic risk for patients with CAD remain challenging as event risk may vary considerably at the individual level. For the effective allocation of limited health resources to patients with the highest risk, new approaches for assessing risk in poor prognosis populations are required. Currently, multiple panels based on the different combinations of clinically available biomarkers exhibit limited prognostic performance and do not provide additional information on molecular targets for therapeutic intervention.^4^, ^5^ Hence, identifying effective biomarkers for improved risk stratification and discovering novel molecular targets involved in the underlying pathological mechanism of CAD should be prioritize.

Death or major adverse cardiovascular event (MACE) is a heterogeneous endpoint associated with a range of lipid metabolic abnormalities. Although traditional clinical lipids are reportedly associated with mortality and left ventricular (LV) dysfunction,^6^, ^7^, ^8^, ^9^ each measure indicates the complex mixture of molecular species which are not sensitive enough to reflect the abundance and complexity of altered lipid metabolism associated with clinical endpoints of CAD.^10^ The widely targeted lipidomic profiling colligates the high throughput of untargeted lipidomics and stability and accuracy of targeted lipidomics, which helps to identify the lipid biomarkers of main outcomes and to clarify the relationship between the key lipids and CAD.

Lipidic biomarkers for predicting clinical endpoints of CAD are few.^11^, ^12^ Ceramides and its different ratios are effective biomarkers for predicting the risk of cardiovascular death in stable CAD.^13^, ^14^, ^15^, ^16^, ^17^ Higher circulating plasma ceramide ratio (C16:0/C24:0) also has an adverse effect on cardiac remodelling.^18^ Sphingolipids, phospholipids, and glycerolipids are significantly related to the risks of cardiovascular events and death in patients with type 2 diabetes.^19^ However, the spectrum of lipid metabolites for predicting death and MACE risks in Chinese patients with CAD is a major concern.

Therefore, we hypothesized that specific lipid metabolites in plasma have a crucial impact on the occurrence of clinical endpoints and LV remodeling in patients with CAD, and that the combination of plasma lipids with traditional factors may improve the predictive power of death and MACE compared with traditional biomarkers only. Herein, we performed a high‐throughput widely targeted lipidomic profiling on 1011 patients with CAD to access the relationships between lipid species and main outcomes as well as LV function. Our results were subsequently validated on 558 patients with CAD enrolled from three hospitals. In brief, we established a powerful predictive model based on novel lipid metabolites and traditional risk factors for estimating future risk of death for patients with CAD.

## 2 MATERIALS AND METHODS

### 2.1 Study populations

**FIGURE 1:**
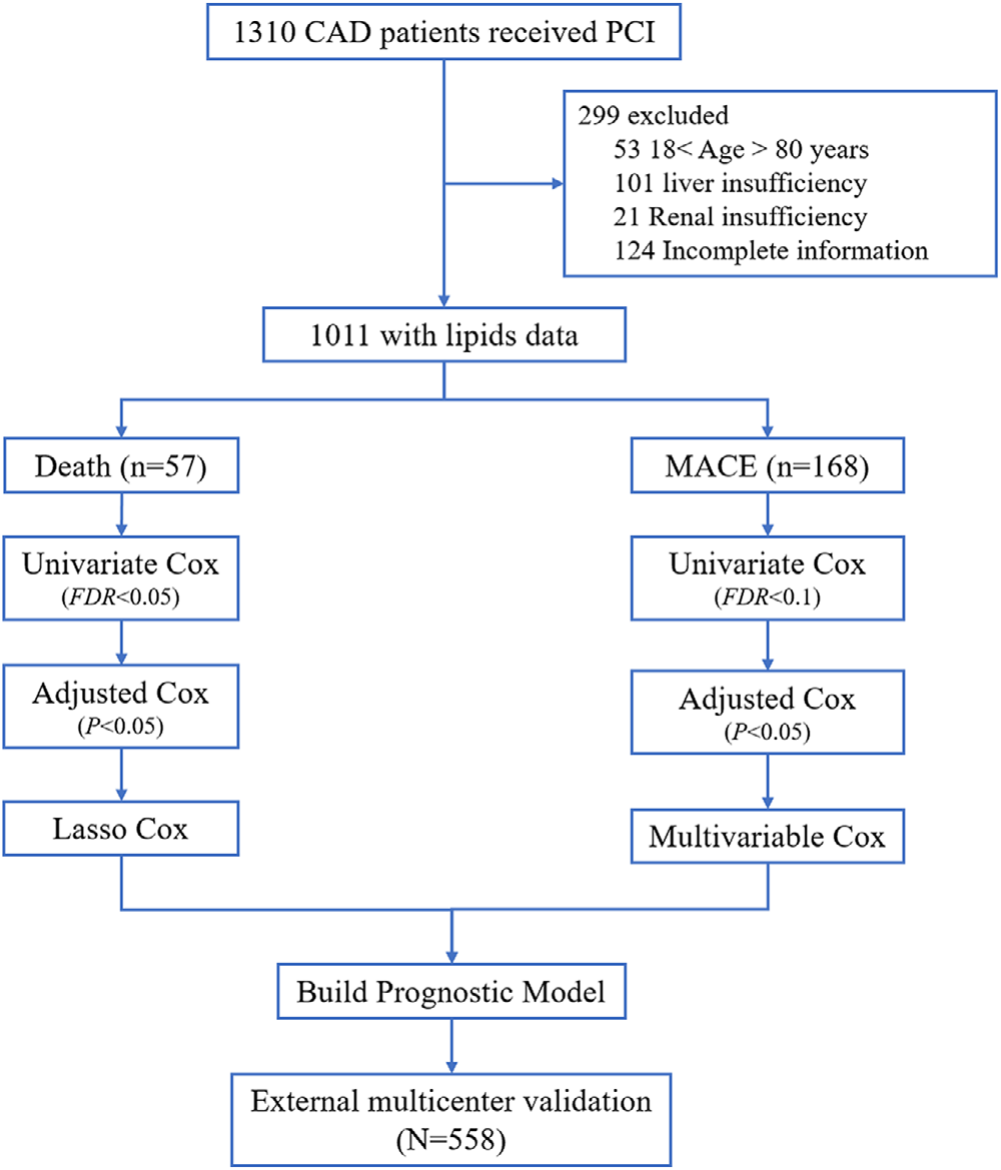
Workflow chart of data generation and analysis

The workflow of this trial is depicted in Figure 1. A total of 1569 patients with CAD from three clinical centers were enrolled and divided into two cohorts. The internal training cohort included 1011 patients who received percutaneous coronary intervention (PCI) treatment from Guangdong Provincial People's Hospital from 2010 to 2013 and were followed up for all‐cause death and MACE up to 5 years. The external validation cohort consisted of 558 patients from three centers (Guangdong Provincial People's Hospital, Xiangya Hospital of Centre‐South University, and First Affiliated Hospital of Sun Yat‐sen University) from September 2017 and followed up until December 2019. The Synergy between PCI with TAXUS and Cardiac Surgery (SYNTAX) score was calculated to assess the severity, and echocardiography was used in determining LV function and structure.

In general, patients with an indication for diagnostic coronary angiography or PCI on account of CAD were enrolled into our study. Individuals with obstruction of ≥50% of the luminal diameter in at least one main coronary artery were diagnosed with CAD. Patients with CAD were further classified into two subgroups of stable CAD and acute coronary syndrome (ACS). Specifically, ACS was diagnosed according to the ACC/AHA guidelines including unstable angina, ST‐elevation myocardial infraction (MI), and Non‐ST elevation MI. Individuals within one of the following criteria were excluded: (1) aged < 18 years or aged > 80 years, (2) the concentration of serum creatinine is more than two times the upper limit of normal (230 μmol/L) or with the history of renal transplantation or dialysis, (3) the concentration of serum transaminase is more than two times the upper limit of normal (80 U/L) or with cirrhosis, (4) during pregnancy or breastfeeding, (5) during the advanced stage of cancer or with the history of haemodialysis; (6) history of thyroid problems, taking antithyroid drugs or thyroid hormone medication in the past week, and (7) lost to follow‐up.

To minimize the impact of food and nutrition on the level of lipid species in plasma, all patients underwent fasting blood sampling in the morning during hospitalization. The whole blood sample was collected in EDTA anticoagulant tube and separated into plasma and hemocyte within 2 h with centrifugation at 1000 *g* about 10 min at 4°C. The plasma was then aliquoted into three cryopreservation tubes and stored at −80°C for future analysis.

### 2.2 Widely targeted lipidomic profiling

In the internal training and external validation sets, the widely targeted lipidomic profiling was performed using Ultra‐Performance Liquid Chromatography Mass Spectrometry (UPLC‐MS/MS) system (UPLC, Shim‐pack UFLC SHIMADZU CBM30A; MS, Applied Biosystems SCIEX 6500+ QTRAP) at Wuhan Metware Biotechnology. Totally, 667 plasma endogenous lipid species consisting of 14 lipid classes/subclasses and 687 lipid species containing 20 lipid classes/subclasses were annotated in the internal training and external validation cohorts, respectively. Totally, 309 identical lipid species were detected in both cohorts. These lipid species mainly include monoglyceride (MG), cholesteryl esters (CE), diacylglycerol (DG), triacylglycerol (TG), phosphatidic acids (PA), phosphatidylcholines (PC), phosphatidylglycerol (PG), phosphatidylserines (PS), phosphatidylethanolamines (PE), lysophosphatidic acids (LPA), lysophosphatidylcholine (LPC), lysophosphatidylethanolamine (LPE), hemolytic serine (LPS), and ceramides (Cer). The ESI full scan mass spectra ion pairs and conditions for tandem mass spectrometry analysis of the lipid species are shown in Supporting information Figure S1 and Table S1, respectively.

The detailed methodology of the lipidomics was consistent with a previous reported study^20^ and supplemented in the Supporting Information, Methods. Briefly, lipid species were extracted from the plasma of CAD patients. First, the sample was thawed on ice. Second, 50 μL of plasma and 1 mL of lipid extraction reagent were pooled into the corresponding numbered centrifuge tube. The mixture was vortexed for 2 min, added with 500 μL of deionized water, vortexed about 1 min, and centrifuged at 12,000 r/min around 10 min. Third, 500 μL of supernatant was absorbed into the numbered centrifuge tube and concentrated after centrifugation. Last, the powder was dissolved with 100 μL of mobile phase B (comprising 10% acetonitrile, 90% isopropanol, 0.04% acetic acid, and 5 mmol/L ammonium formate), and the dissolving solution was then used for UPLC‐MS/MS analysis.

The calibration and quality control (QC) samples were prepared with the mixed plasma of subjects prior to sample analysis. Every 10 samples to be analyzed were separated by one QC sample for the duration of the detection to monitor repeatability during the analysis. The high overlaps of the total ion flow between different QC samples, that is, the retention time and peak strength are consistent, indicates that the signal stability of the mass spectrum is good at different times. Qualitative analysis of the MS and MS/MS mass spectrometric data was performed on the basis of the homemade database Metware database (MWDB) and the public database of metabolite information. The lipid metabolite structural analysis mainly referred to MassBank, KNAPSAcK, HMDB, Lipidmaps, and METLIN database. Analyst 1.6.3 software (AB Sciex) was used to process the raw mass spectrometry data.

### 2.3 Statistical analysis

For the baseline characteristics of both cohorts, the categorical variables are expressed as counts (percentages), and continuous variables are expressed as means and standard deviation (mean ± SD). Prior to association analyses, the raw data were corrected with the quality control‐robust LOESS signal correction algorithm for the minimization of the batch effect. Pareto scaling was used in interpreting hazard ratio (HR).

Univariate Cox regression analysis was used to recognize clinical characteristics and lipids associated with clinical endpoints and estimate HRs and 95% confidence intervals (CIs). The relationships between baseline characteristics and lipid species against ACS versus stable CAD were accessed by logistic regression analysis with results presented as odds ratio (OR) and 95% CI. Linear regression analysis was used in identifying baseline characteristics and lipids related to LV function. The significant characteristics were used as covariates in adjusted analysis. Potential characteristics included age, sex, comorbidities, drugs, SYNTAX scores, and renal and hepatic dysfunction. A two‐tailed *P* value of .05 was used to indicate statistical significance, and false discovery rate (FDR) was used in correcting the number of lipid species for multiple hypothesis testing. Backward stepwise process based on the Akaike information criterion (AIC) was used in multivariate Cox regression analysis for feature subset selection.

To identify conditional correlations between prognostic lipid species and traditional CAD factors, partial correlation coefficients were calculated for each lipid species and conventional lipids with visualization in Cytoscape. Linear regression analysis was employed to calculate the correlation between the HR of death and structural characteristics of individual lipid species. Pathway enrichment analysis was conducted on prognostic lipid species (adjusted *P* < .05) with Fisher's exact test by MetaboAnalyst 4.0.

The prognostic model of death was constructed by least absolute shrinkage and selection operator (lasso) Cox regression analysis (“glmnet” package). Variables with FDR of < 0.05 in univariate and adjusted *P* of < .05 in adjusted Cox regression analysis were employed into the lasso Cox regression to screen the most powerful predictive features. This procedure was executed with a 10‐fold cross‐validation framework (200 repeats, Supporting information Figure S2), and the lipid independently associated with death should be selected over 180 times. For MACE, variables with adjusted *P *< .05 were included for the formulation of prognostic models. Variables retained in the model were considered independent lipid species (Figure 1).

To evaluate the prediction efficiency of the multivariable models, we calculated the risk estimate of individuals for clinical endpoints on the basis of regression coefficients according to the following formula: *h*(t,X) = *h0*(*t*)exp(*β*1 × 1 + *β*2 × 2 +⋯+ *β*i*X*i), where *h*0(t) is the baseline hazard rate at specific time t (“survival” package), *β* is the regression coefficients, and *X*i is the selected marker. Time‐dependent receiver‐operating characteristic (ROC) analysis, continuous net reclassification improvement (NRI), and integrated discrimination improvement (IDI) were used in assessing the discrimination of the predictive model for death and MACE. The external validation of prognostic models was performed in the multicentre cohort based on the individually calculated hazard estimates, and hazard stratification was presented by Kaplan‐Meier curves between low (<Q1), middle (≥Ql and ≤Q3), and high (>Q3) hazard estimates.

Statistical analyses were executed by GraphPad Prism 8 and R. The detailed methods are presented in Supporting Information, Methods.

## 3 RESULTS

### 3.1 Baseline characteristics

Demographic characteristics and their impact on clinical endpoints and ACS are summarized in Tables 1 and 2. Totally, patients with ACS accounted for 50.1% in the internal training cohort and 38.7% in the external validation cohort. Individuals who died during the follow‐up tended to be older, accompanied with higher levels of aspartate aminotransferase (AST), brain natriuretic peptide (BNP), and SYNTAX score but lower estimated glomerular filtration rate. Individuals who experienced MACE had higher levels of AST, BNP, and SYNTAX score. In addition, patients with ACS tended to have higher levels of alanine aminotransferase, AST, glucose, BNP, and SYNTAX score but lower level of apolipoprotein A. They had a history of angiotensin‐converting enzyme inhibitor medication as well. Taking statin showed no effect on clinical outcomes and ACS in both cohorts. The associations between baseline characteristics with LV ejection fraction (LVEF) and LV mass index (LVMI) are summarized in Supporting information Table S2.

**TABLE 1:** Baseline characteristics and effects on clinical endpoints and ACS in the internal training cohort

	Value N (%) or	Death		MACE		ACS	
Characteristics	mean ± SD	HR (95% CI)	*P* Value	HR (95% CI)	*P* value	OR (95% CI)	*P* value
Demographic data							
Age	63.01 ± 10.07	1.08 (1.05‐1.12)	1.49 × 10^‐6^	1.01 (0.99‐1.03)	1.92 × 10^‐1^	0.99 (0.98‐1.00)	2.27 × 10^‐1^
SEX (male)	805 (79.62)	1.00 (0.52‐1.93)	9.98 × 10^‐1^	1.00 (0.69‐1.47)	9.87 × 10^‐1^	1.20 (0.88‐1.63)	2.54 × 10^‐1^
BMI, kg/m²	24.27 ± 4.82	0.90 (0.81‐1.00)	4.64 × 10^‐2^	0.97 (0.92‐1.02)	2.51 × 10^‐1^	0.95 (0.91‐0.99)	1.37 × 10^‐2^
Comorbidities							
Arrhythmia	88 (8.72)	1.99 (0.98‐4.05)	5.84 × 10^‐2^	1.80 (1.16‐2.79)	9.06 × 10^‐3^	0.81(0.52‐1.26)	3.47 × 10^‐1^
Diabetes	277 (27.45)	2.41 (1.43‐4.06)	9.11 × 10^‐4^	1.59 (1.16‐2.17)	3.69 × 10^‐3^	1.08(0.82‐1.42)	5.91 × 10^‐1^
Heart failure	87 (8.62)	3.60 (1.97‐6.58)	3.20 × 10^‐5^	2.65 (1.79‐3.91)	9.73 × 10^‐7^	1.31(0.84‐2.04)	2.37 × 10^‐1^
Hypertension	605 (59.90)	0.99 (0.58‐1.68)	9.70 × 10^‐1^	1.28 (0.93‐1.75)	1.31 × 10^‐1^	0.88(0.68‐1.13)	3.23 × 10^‐1^
Hyperlipidemia	112 (11.09)	0.33 (0.08‐1.34)	1.20 × 10^‐1^	0.96 (0.58‐1.59)	8.83 × 10^‐1^	0.81(0.54‐1.20)	2.96 × 10^‐1^
Biochemical measurements							
ALT, U/L	27.62 ± 15.08	1.01 (0.99‐1.03)	1.94 × 10^‐1^	1.01 (0.99‐1.02)	3.55 × 10^‐1^	1.02 (1.01‐1.03)	3.90 × 10^‐4^
AST, U/L	26.83 ± 12.13	1.03 (1.01‐1.05)	4.48 × 10^‐3^	1.02 (1.00‐1.03)	1.65 × 10^‐2^	1.03 (1.01‐1.04)	3.89 × 10^‐5^
eGFR, mL/min/1.73 m²	95.02 ± 74.57	0.98 (0.97‐0.99)	2.83 × 10^‐3^	1.00 (0.99‐1.00)	2.49 × 10^‐1^	1.00 (1.00‐1.00)	1.02 × 10^‐1^
CK, U/L	112.76 ± 112.54	1.00 (1.00‐1.00)	4.72 × 10^‐1^	1.00 (1.00‐1.00)	9.36 × 10^‐1^	1.00 (1.00‐1.00)	7.61 × 10^‐1^
CKMB, U/L	7.54 ± 5.92	1.01 (0.97‐1.06)	6.61 × 10^‐1^	1.02 (0.99‐1.04)	1.35 × 10^‐1^	1.02 (1.00‐1.05)	6.05 × 10^‐2^
CHOL, mmol/L	4.28 ± 1.12	1.00 (0.79‐1.26)	9.91 × 10^‐1^	1.02 (0.90‐1.17)	7.27 × 10^‐1^	0.94 (0.83‐1.05)	2.44 × 10^‐1^
LDLC, mmol/L	2.58 ± 0.90	0.91 (0.68‐1.23)	5.56 × 10^‐1^	0.99 (0.83‐1.17)	8.74 × 10^‐1^	1.00 (0.87‐1.15)	9.69 × 10^‐1^
HDLC, mmol/L	0.97 ± 0.26	0.49 (0.17‐1.41)	1.86 × 10^‐1^	0.76 (0.42‐1.38)	3.64 × 10^‐1^	0.43 (0.26‐0.70)	8.76 × 10^‐4^
GLUC, mmol/L	6.70 ± 2.69	1.08 (1.01‐1.17)	3.64 × 10^‐2^	1.03 (0.98‐1.08)	2.94 × 10^‐1^	1.06 (1.02‐1.12)	1.04 × 10^‐2^
Lpa, mg/L	304.64 ± 321.15	1.00 (1.00‐1.00)	1.26 × 10^‐1^	1.00 (1.00‐1.00)	3.71 × 10^‐1^	1.00 (1.00‐1.00)	6.52 × 10^‐1^
APOA, g/L	1.05 ± 0.28	0.23 (0.06‐0.81)	2.25 × 10^‐2^	0.62 (0.33‐1.16)	1.34 × 10^‐1^	0.48 (0.28‐0.79)	4.71 × 10^‐3^
BNP, pg/mL	5.45 ± 1.63	1.48 (1.21‐1.82)	1.64 × 10^‐4^	1.14 (1.01‐1.28)	2.95 × 10^‐2^	2.27 (1.81‐2.87)	4.35 × 10^‐12^
TRIG, mmol/L	1.61 ± 1.15	1.07 (0.92‐1.26)	3.78 × 10^‐1^	1.08 (0.98‐1.18)	1.34 × 10^‐1^	1.00 (0.89‐1.11)	9.55 × 10^‐1^
Medication							
β‐blockers	895 (88.70)	1.06 (0.45‐2.46)	8.97 × 10^‐1^	1.81 (0.98‐3.33)	5.81 × 10^‐2^	1.23(0.83‐1.82)	3.04 × 10^‐1^
ACEIs	622 (61.65)	0.80 (0.47‐1.36)	4.07 × 10^‐1^	1.22 (0.88‐1.70)	2.22 × 10^‐1^	1.47 (1.14‐1.90)	2.86 × 10^‐3^
CCBs	278 (27.55)	2.15 (1.28‐3.62)	4.05 × 10^‐3^	1.71 (1.26‐2.33)	6.77 × 10^‐4^	0.78 (0.59‐1.03)	8.05 × 10^‐2^
PPIs	490 (48.56)	1.73 (1.01‐2.96)	4.75 × 10^‐2^	1.81 (1.32‐2.47)	2.21 × 10^‐4^	1.12 (0.88‐1.44)	3.60 × 10^‐1^
Statin	861 (85.8)	1.54 (0.61‐3.86)	3.58 × 10^‐1^	0.97 (0.62‐1.53)	9.08 × 10^‐1^	1.02 (0.71‐1.45)	9.28 × 10^‐1^
SYNTAX score	16.35 ± 10.67	1.03 (1.00‐1.05)	2.51 × 10^‐2^	1.02 (1.01‐1.03)	5.24 × 10^‐3^	1.02 (1.01‐1.03)	2.76 × 10^‐3^

HRs (95% CI) were calculated by applying a Cox regression analysis and ORs (95% CI) were calculated by applying a logistic regression analysis. Variables with *P* < .05 were included into the multivariable models as covariates.

ACEIs, angiotensin converting enzyme inhibitors; ALT, alanine aminotransferase; APOA, apolipoprotein a; AST, aspartate aminotransferase; BMI, body mass index; BNP, B‐type natriuretic peptide; CCBs, calcium channel blockers; CK, creatine kinase; CKMB, creatine kinase MB; CHOL, cholesterol; eGFR, estimated glomerular filtration rate; GLUC, glucose; HDLC, high‐density lipoprotein cholesterol; HR, hazard ratio; Lpa, lipoprotein (a); LDLC, low‐density lipoprotein cholesterol; PPIs, proton pump inhibitors; SD, standard deviation; SYNTAX score, Synergy between PCI with TAXUS and Cardiac Surgery score; TRIG, triglyceride.

**TABLE 2:** Baseline characteristics and the effects on clinical endpoints and ACS in the external validation cohort

	Value N (%) or	Death		MACE		ACS	
Characteristics	mean ± SD	HR (95% CI)	*P* value	HR (95% CI)	*P* value	OR (95% CI)	*P* value
Demographic data							
Age	62.15 ± 10.24	1.06 (1.01‐1.12)	1.10 × 10^‐2^	1.04 (1.01‐1.07)	2.11 × 10^‐2^	1.01 (0.99‐1.02)	4.08 × 10^‐1^
SEX (male)	426 (74.21)	1.12 (0.41‐3.05)	8.29 × 10^‐1^	0.73 (0.38‐1.37)	3.22 × 10^‐1^	0.68 (0.46‐1.00)	5.14 × 10^‐2^
BMI, kg/m²	24.04 ± 3.35	0.80 (0.24‐1.40)	6.80 × 10^‐2^	0.98 (0.87‐1.12)	7.94 × 10^‐1^	1.00 (0.93‐1.07)	9.98 × 10^‐1^
Comorbidities							
Arrhythmia	52 (9.15)	2.34 (0.78‐7.00)	1.29 × 10^‐1^	1.93 (0.86‐4.34)	1.12 × 10^‐1^	0.85 (0.46‐1.53)	5.90 × 10^‐1^
Diabetes	164 (28.87)	1.21 (0.49‐2.99)	6.87 × 10^‐1^	1.23 (0.66‐2.29)	5.06 × 10^‐1^	1.08 (0.74‐1.57)	6.76 × 10^‐1^
Heart failure	257 (45.25)	1.88 (0.78‐4.53)	1.61 × 10^‐1^	1.13 (0.63‐2.03)	6.80 × 10^‐1^	1.99 (1.41‐2.82)	1.03 × 10^‐4^
Hypertension	344 (60.67)	2.09 (0.76‐5.70)	1.51 × 10^‐1^	2.06 (1.04‐4.06)	3.76 × 10^‐2^	1.19 (0.83‐1.69)	3.45 × 10^‐1^
Hyperlipidemia	76 (13.38)	0.62 (0.14‐2.65)	5.17 × 10^‐1^	0.75 (0.30‐1.91)	5.52 × 10^‐1^	1.35 (0.82‐2.21)	2.29 × 10^‐1^
Biochemical							
Measurements							
ALT, U/L	27.84 ± 24.74	1.01 (0.99‐1.02)	2.97 × 10^‐1^	1.00 (0.99‐1.01)	9.49 × 10^‐1^	1.01 (1.00‐1.02)	3.53 × 10^‐3^
AST, U/L	32.18 ± 55.09	1.00 (1.00‐1.01)	9.92 × 10^‐4^	1.00 (1.00‐1.01)	1.54 × 10^‐2^	1.02 (1.01‐1.03)	4.41 × 10^‐4^
eGFR, ml/min/1.73 m²	93.29 ± 119.07	0.97 (0.95‐0.98)	9.18 × 10^‐5^	0.98 (0.97‐0.99)	5.17 × 10^‐4^	0.99 (0.99‐1.00)	1.09 × 10^‐1^
CK, U/L	162.54 ± 449.87	1.00 (1.00‐1.00)	1.66 × 10^‐3^	1.00 (1.00‐1.00)	8.84 × 10^‐3^	1.00 (1.00‐1.00)	1.07 × 10^‐2^
CKMB, U/L	19.15 ± 52.44	0.98 (0.94‐1.03)	5.23 × 10^‐1^	1.00 (0.99‐1.01)	8.83 × 10^‐1^	1.04 (1.02‐1.07)	7.23 × 10^‐5^
CHOL, mmol/L	4.30 ± 1.76	0.79 (0.52‐1.20)	2.62 × 10^‐1^	0.99 (0.81‐1.21)	9.31 × 10^‐1^	0.96 (0.85‐1.07)	5.13 × 10^‐1^
LDLC, mmol/L	2.72 ± 1.01	0.75 (0.43‐1.32)	3.19 × 10^‐1^	1.12 (0.86‐1.46)	4.18 × 10^‐1^	0.99 (0.83‐1.18)	9.37 × 10^‐1^
HDLC, mmol/L	0.99 ± 0.25	0.37 (0.05‐2.82)	3.39 × 10^‐1^	1.18 (0.37‐3.84)	7.77 × 10^‐1^	0.63 (0.31‐1.28)	2.11 × 10^‐1^
GLUC, mmol/L	6.02 ± 2.20	1.13 (0.98‐1.31)	8.89 × 10^‐2^	1.07 (0.96‐1.21)	2.28 × 10^‐1^	1.10 (1.02‐1.20)	1.83 × 10^‐2^
Lpa, mg/L	285.29 ± 324.06	1.00 (1.00‐1.00)	9.95 × 10^‐1^	1.00 (1.00‐1.00)	6.42 × 10^‐1^	1.00 (1.00‐1.00)	2.03 × 10^‐1^
APOA, g/L	1.15 ± 0.25	0.42 (0.05‐3.63)	4.28 × 10^‐1^	0.51 (0.11‐2.38)	3.91 × 10^‐1^	0.31 (0.10‐0.87)	3.30 × 10^‐2^
BNP, pg/mL	2.26 ± 0.75	4.32 (2.63‐7.10)	7.26 × 10^‐9^	2.12 (1.49‐3.01)	2.90 × 10^‐5^	1.95 (1.53‐2.50)	1.06 × 10^‐7^
TRIG, mmol/L	1.85 ± 1.84	0.69 (0.37‐1.29)	2.46 × 10^‐1^	0.95 (0.74‐1.22)	6.89 × 10^‐1^	1.20 (1.04‐1.40)	1.91 × 10^‐2^
Medication							
β‐blockers	483 (84.29)	0.60 (0.22‐1.63)	3.16 × 10^‐1^	1.02 (0.43‐2.44)	9.66 × 10^‐1^	1.44 (0.89‐2.39)	1.43 × 10^‐1^
ACEIs	287 (50.00)	0.47 (0.19‐1.17)	1.05 × 10^‐1^	1.1 0(0.59‐2.06)	9.58 × 10^‐1^	1.74 (1.23‐2.46)	1.69 × 10^‐3^
CCBs	168 (30.11)	1.47 (0.61‐3.56)	3.87 × 10^‐1^	1.18 (0.61‐2.32)	6.85 × 10^‐1^	0.59 (0.40‐0.88)	1.03 × 10^‐2^
PPIs	388 (67.60)	0.84 (0.35‐2.03)	6.95 × 10^‐1^	0.99 (0.50‐1.95)	5.17 × 10^‐1^	0.62 (0.43‐0.89)	8.85 × 10^‐3^
Statin	499 (91.2)	0.48 (0.14‐1.64)	2.41 × 10^‐1^	0.87 (0.31‐2.44)	7.92 × 10^‐1^	1.77 (0.94‐3.55)	9.03 × 10^‐2^
SYNTAX score	16.59 ± 13.18	1.04 (1.00‐1.08)	4.27 × 10^‐2^	1.04 (1.01‐1.06)	9.87 × 10^‐4^	1.03 (1.01‐1.04)	3.47 × 10^‐4^

HRs (95% CI) were calculated by applying a Cox regression analysis and ORs (95%CI) were calculated by applying a logistic regression analysis. Variables with *P* < .05 were entered into the multivariable models as covariates. Abbreviations as in Table 1.

Notably, we used the SYNTAX score to indicate the severity of patients with CAD. SYNTAX score independently predicts MACE and long‐term prognosis in patients with stable CAD who received revascularisation.^21^, ^22^ Taking the internal training cohort with a large number of patients for a detailed illustration (Table 1), we found that SYNTAX score was positively related to death (HR (95% CI): 1.03 (1.00–1.05); *P* = .0251) and MACE (HR (95% CI):1.02 (1.01–1.03); *P* = .0052) risks. Patients with plaque rapture tended to have higher SYNTAX score against those with stable CAD. Furthermore, patients with relatively poor cardiac function who presented reduced LVEF (estimate ± SE: −0.24 ± 0.04; *P* = 7.23E‐11) and increased LVMI (estimate ± SE: 0.32 ± 0.13; *P* = .0125) had higher SYNTAX score (Supporting information Table S2). Thus, the SYNTAX score was used to correct the influences of CAD severity on prognosis.

### 3.2 Relationships between lipid species and clinical outcomes

Among the 667 targeted lipid species detected in the internal training cohort, 85 different lipid species were significantly related to all‐cause death (FDR < 0.05), of which 78 lipid species were still associated with the incident death after adjustment for potential confounders (*P* < .05, Supporting information Table S3). These lipid species mainly belonged to sphingolipids, glycerolphospholipid, monoglyceride, diglyceride, and triglyceride. Pathway analysis revealed that sphingolipids metabolism, glycerolphospholipid metabolism, and glycosylphosphatidylinositol‐anchor biosynthesis were the top three disturbed metabolic pathways in patients with death risk (Supporting information Figure S3A and Table S6). In addition, five differential lipid species were significantly correlated with MACE after potential confounders were adjusted (Supporting information Table S4). Specifically, LPC(18:2/0:0) was inversely associated with death and MACE risks, and Cer(d18:1/20:1), HexCer(d18:1/18:1), and HexCer(d18:1/20:1) were positively associated with death and MACE risks.

**FIGURE 2:**
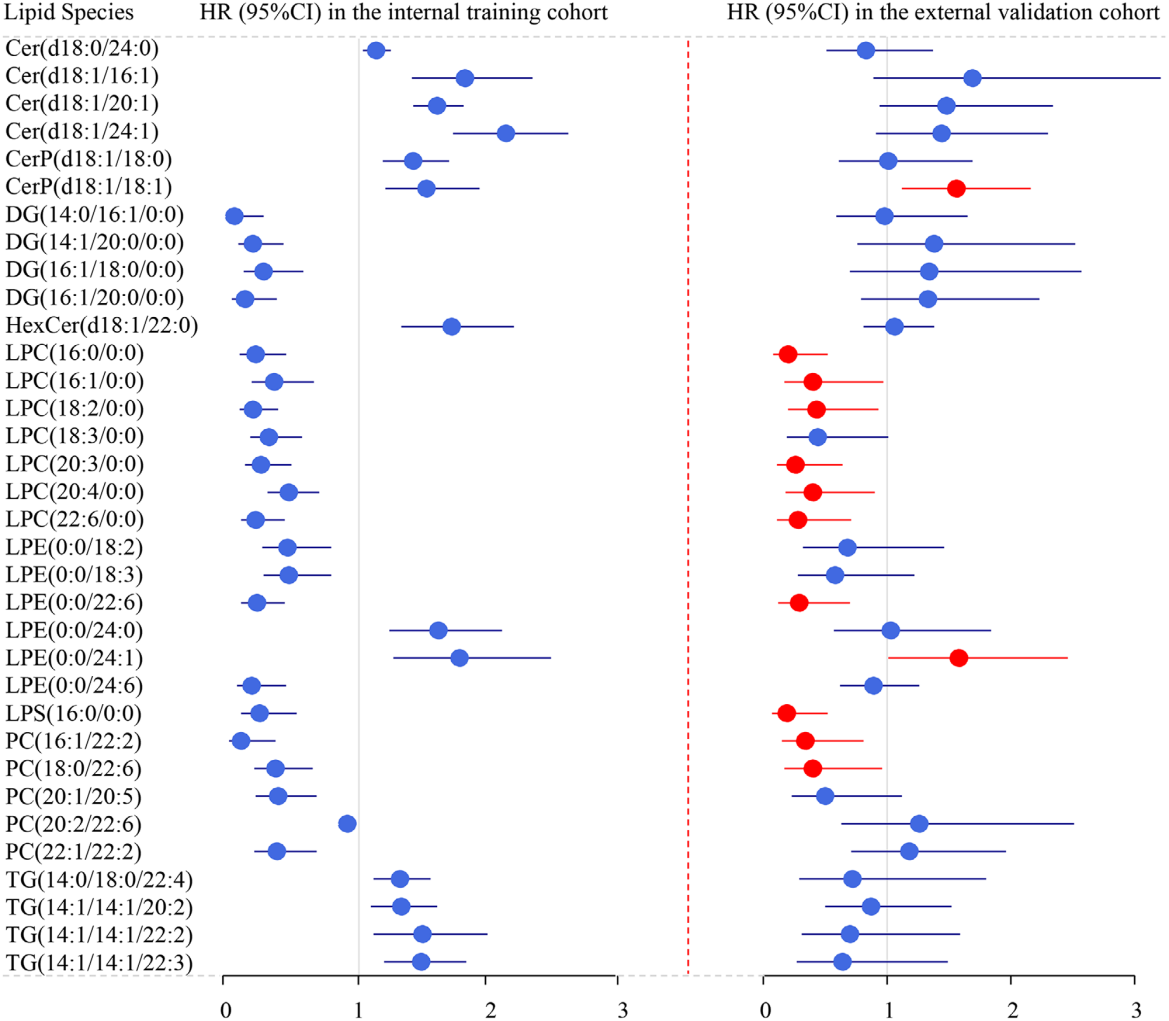
Forest plot of the hazard ratio on lipid species related to death. Forest plot of the hazard ratio of the death of 34 lipid species in the internal training (left) and external validation cohorts (right). HRs (circles) indicate the risk of change in each lipid species of 1 SD for ease of comparison. Bars represent 95% confidence intervals. Red‐coded circles and bars indicate that the lipid species were replicated in the external validation cohort. Cer, ceramide; DG, diglyceride; HexCer, monohexosylceramide; LPC, lysophosphatidylcholine; LPE, lysophosphatidylethanolamine; LPS, lysophosphatidylserine; PC, phosphatidylcholine; TG, triglyceride

In the external validation cohort, only 34 of the 78 lipid species associated with death risk were repeatedly detected owing to the improvement in detection platform. Finally, 12 lipid metabolites showed statistical difference with death in univariate Cox analysis, and three lipid species (LPC(16:0/0:0), LPS(16:0/0:0), and LPC(20:3/0:0)) showed statistical difference in adjusted Cox analysis (Figure 2 and Supporting information Table S5). None of the five lipid species were repeatedly correlated with the MACE risk in the external validation cohort.

**FIGURE 3:**
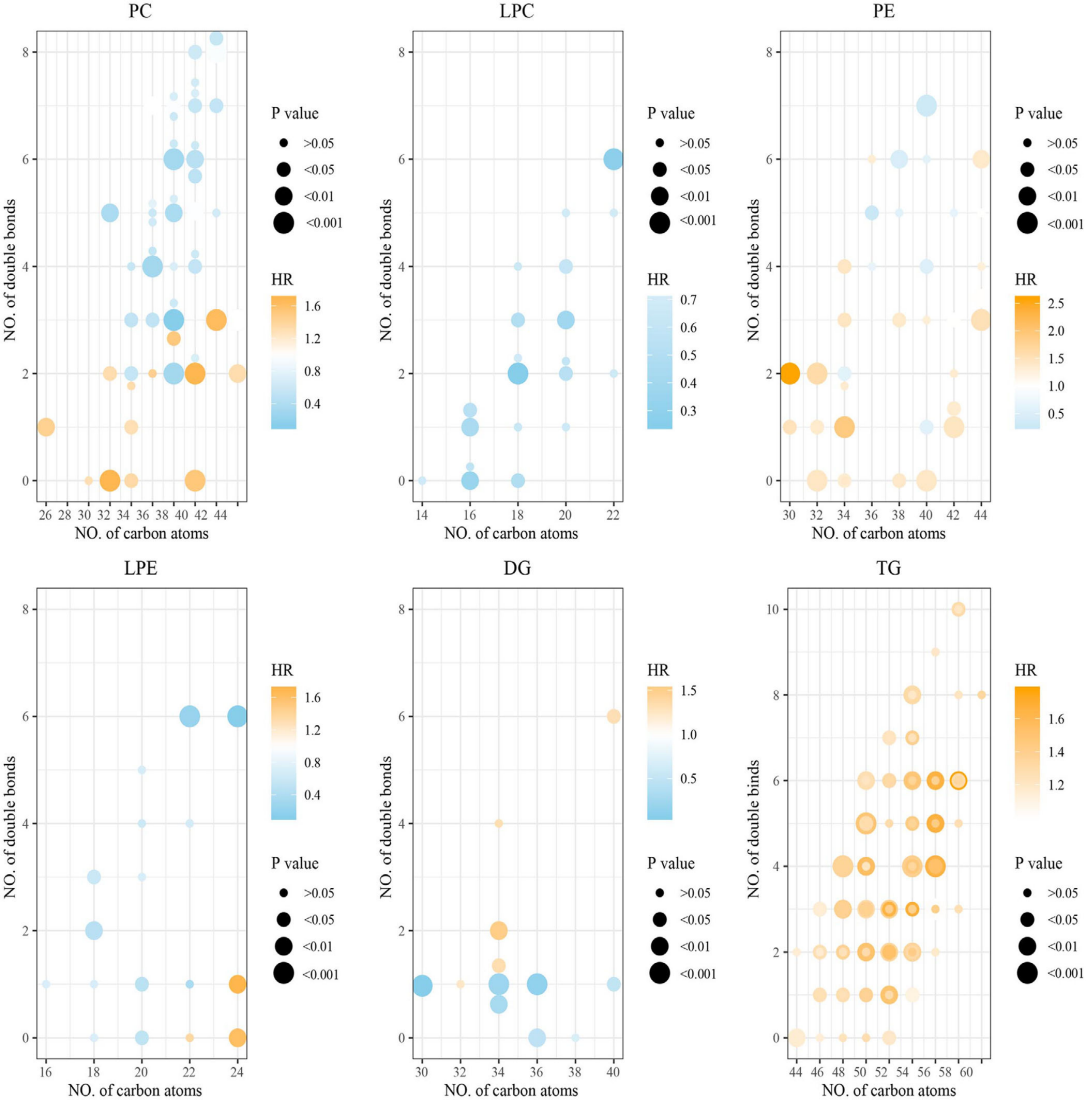
Bubble charts of the association of 266 lipid species with death risk in the internal training cohort. Each filled circle represents an individual lipid metabolite, and six lipid subclasses are prensented by 2D bubble plots according to the number of total carbon atoms (x axis) and the number of double bonds (y axis). The shade of the color indicates the magnitude of HR. The radius of the circle indicates the significance level (Legend). To increase visibility, lipid species with equal numbers of carbon atoms and double bonds were pulled apart vertically, except DG. HRs were calculated byCox regression adjusting for age, AST, DM, HF, eGFR, GLUC, CCB, PPI, and SYNTAX score. DG, diglyceride; LPC, lysophosphatidylcholine; LPE, lysophosphatidylethanolamine; PC, phosphatidylcholine; PE, phosphatidylethanolamine; TG, triglyceride

Linear regression analyses showed that the HR of death was inversely related to the number of double bonds (estimate [SE], −0.09 [0.02]; *P* = 6.52E‐05) and carbon atoms (estimate [SE], −0.03 [0.01]; *P* = .098) in the acyl chains of PC. The number of double bonds (estimate [SE], −0.12 [0.03]; *P* = 9.28E‐04) and carbon atoms (estimate [SE], −0.04 [0.02]; *P *= .046) of PE were significantly negatively correlated with the HR of death. Only the number of double bonds (estimate [SE], −0.11 [0.04]; *P* = .026) was correlated with death risk in LPE. LPC, DG, and TG did not show statistical difference (Figure 3). Similarly, the number of double bonds of PC was negatively related to death risk (estimate [SE],–0.12 [0.06]; *P* = .041) and the number of carbon atoms of PE was negatively correlated with HR (estimate [SE], −0.07 [0.03]; *P* = .022) in the multicentre cohort (Supporting information Figure S4). These results underline the importance for mechanistic research that characterizes the role of individual lipid species on disease status.

### 3.3 Prognostic lipid species correlations with conventional CAD biomarkers

**FIGURE 4:**
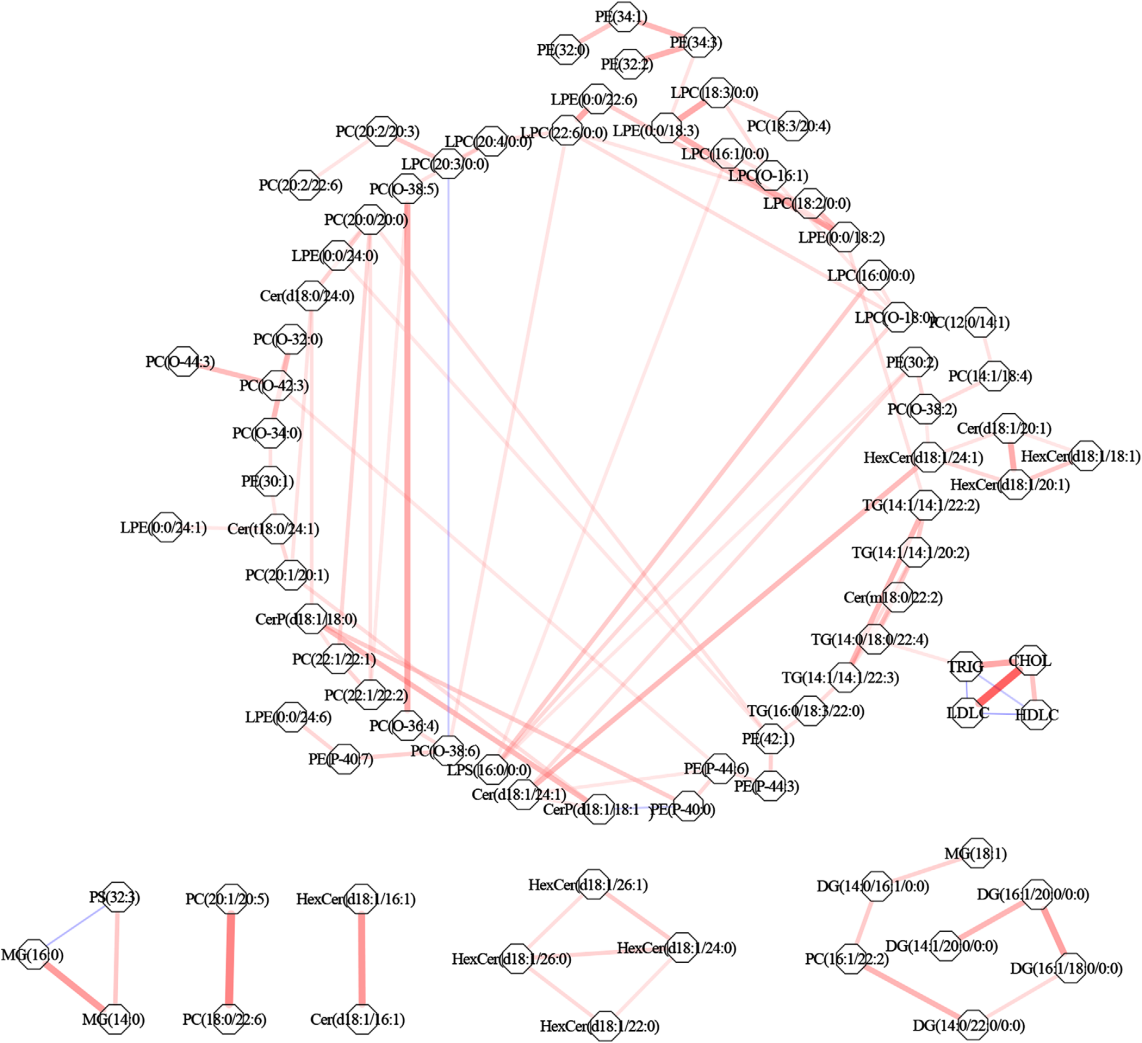
Partial correlations between prognostic lipid species and conventional lipid markers in patients with CAD. Conditioned on the presence of all other analytes (*r* ≥ 0.20). Analytes are represented by nodes (grey hexagons) and conditional correlations by edges (lines). Pink lines indicate positive correlations. Blue lines indicate inverse correlations. Line width represents the strength of the conditional correlation. The lack of a line indicates the absence of a detectable relationship above the threshold. CHOL, cholesterol; HDLC, high density lipoprotein cholesterol; LDLC, low density lipoprotein cholesterol; TRIG, triglyceride

To investigate the correlation between prognostic lipid species and traditional markers of CAD risks, the partial correlation coefficients of 78 prognostic lipids and LDLC, HDLC, CHOL, and TRIG (Figure 4) were calculated. All relationships were defined with the existence of other influencing factors (*r* ≥ 0.20), indicating direct relations rather than the influence by other components. Our results illustrated that these lipids are related to each other in a single and interconnected network, whereas their correlations with conventional CAD factors were feeble (*r* < 0.20) except for TG(14:0/18:0/22:4). The most powerful positive relationships (red lines) were between PC(18:0/22:6) and PC(20:1/20:5), Cer(d18:1/16:1) and HexCer(d18:1/16:1) and MG(14:0) and MG(16:0). As expected, a forceful positive correlation was found between LDLC and CHOL. Our results indicated that sphingolipids and glycerophospholipid are mainly independent of conventional CAD lipids and thus present novel insights into the progression of disease.

### 3.4 Generating optimized prognostic models for clinical endpoints

**FIGURE 5:**
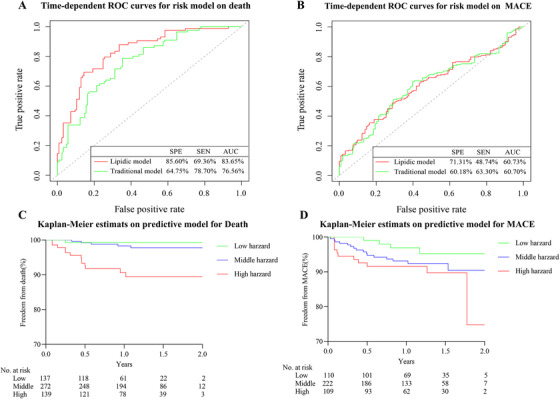
Prognostic lipid species of death and MACE independent of traditional risk factors. ROC curves for all‐cause mortality (A) and ROC curves for MACE (B). ROC curve = receiver–operating characteristic curve; SPE = specificity; SEN = sensitivity. The Kaplan–Meier curves of the predictive model for death (C) and MACE (D) in the external validation cohort

We developed two prognostic models for predicting 5‐year death risk. In the first model (termed traditional model), we inputted nine significant covariates for death in Table 1 into multivariate Cox regression model, and five (age, DM, HF, AST, and SYNTAX score) variables were retained in the final model with minimal AIC. In the second model (termed lipidic model), our input included the aforementioned traditional variables and 78 lipid species significantly associated with death. The optimal model consisting 10 independent lipid species and two traditional risk markers (age and AST) were obtained by lasso Cox regression (200 repeats, Table 3). Lasso Cox analysis showed that Cer(d18:1/20:1), Cer(d18:1/24:1), PE(30:2), PE(32:0), and PE(32:2) were independent risk lipid metabolites for death, whereas LPC(18:2/0:0), LPE(0:0/24:6), PC(16:1/22:2), PC(O‐36:4), and PC(O‐38:2) were independent protective lipid metabolites for death. The incorporation of 10 lipid species to classical markers for the prognosis of 5‐year death risk yielded a remarkable higher discrimination than the traditional model with increased area under the curve (AUC) from 76.56 to 83.65% (Figure 5A), continuous NRI of 0.634 (95% CI, 0.408‐0.753) and IDI of 0.131 (95% CI, 0.074–0.281; Table 4). This model was subsequently used in estimating two‐year survival probabilities from death of each patient in the multicentre cohort and was successfully used in differentiating patients with low, medium, and high risks of death (*P* < .0001; Figure 5C).

**TABLE 3:** Features in the predictive model of death risk selected by 10‐fold cross‐validation lasso Cox regression analysis (200 repeats)

Terms	Coefficient (*β*)	HR	N
Age	.019	1.020	199
AST	.005	1.005	184
Cer(d18:1/20:1)	.156	1.169	200
Cer(d18:1/24:1)	.095	1.099	200
LPC(18:2/0:0)	−.205	0.815	193
LPE(0:0/24:6)	−.233	0.792	200
PC(16:1/22:2)	−.140	0.870	191
PC(O‐36:4)	−.156	0.855	199
PC(O‐38:2)	−.352	0.703	200
PE(30:2)	.401	1.493	200
PE(32:0)	.114	1.121	200
PE(32:2)	.131	1.140	200

The regression coefficients of death were calculated by averaging the coefficients obtained from 200 times lasso Cox analyses, and the HR was calculated by exp(β). The 12 markers were obtained using a lasso Cox analysis in which we required selected markers to appear over 180 times out of a total of 200 repetitions.

Cer, ceramide; HR, Hazard Ratio; LPC, lysophosphatidylcholine; LPE, lysophosphatidylethanolamine; PC, phosphatidylcholine; PC(O), alkylphosphatidylcholine; PE, phosphatidylethanolamines;

**TABLE 4:** Model performance measures (95% CIs) for 5‐year risk in the internal training cohort

Feature	AUC	IDI	Continuous NRI
Prediction of death			
Lipidic model^a^	0.8365		
Traditional model^b^	0.7656	0.131 (0.074‐0.281)	0.634 (0.408‐0.753)
Prediction of MACE			
Lipidic model^c^	0.6073		
Traditional model^d^	0.6070	0.015 (–0.001‐0.039)	0.152 (0.007‐0.273)

^a^ Lipidic model for death based on: Cer(d18:1/20:1), Cer(d18:1/24:1), LPC(18:2/0:0), LPE(0:0/24:6), PC(16:1/22:2), PC(O‐36:4), PC(O‐38:2), PE(30:2), PE(32:0), PE(32:2), age and aspartate aminotransferase.

^b^ Traditional model for death based on: age, diabetes mellitus, and heart failure status, aspartate aminotransferase and SYNTAX score.

^c^ Lipidic model for MACE based on: Cer(d18:1/20:1), and LPC(20:0/0:0), arrhythmia, diabetes mellitus, and heart failure status, SYNTAX score, calcium channel blockers, proton pump inhibitors.

^d^ Traditional model for MACE based on: arrhythmia, diabetes mellitus, and heart failure status, SYNTAX score, calcium channel blockers, proton pump inhibitors and aspartate aminotransferase.

Two prognostic models for MACE were developed using multivariate Cox regression with minimal AIC. The traditional model consisted of seven significant covariates for MACE, and the lipidic model contained six traditional biomarkers (arrhythmia, HF, DM, CCB, PPI, and SYNTAX score) and two lipid species, namely, Cer(d18:1/20:1) and LPC(20:0/0:0). Multivariate Cox regression analysis revealed that Cer(d18:1/20:1) (HR, 1.14; 95% CI, 1.02–1.28; *P* = 2.56E‐02) was the risk lipid predictor for MACE, whereas LPC(20:0/0:0) (HR, 0.65; 95% CI, 0.48–0.88; *P* = 4.60E‐03) was the protective lipid metabolite for MACE (Supporting information Table S7). The ROC curves of MACE are plotted in Figure 5B. The prognostic efficiency of lipidic model showed little improvement than traditional model with AUC from 60.70 to 60.73%, continuous NRI of 0.152 (95% CI, 0.007–0.273) and IDI of 0.015 (95% CI, –0.001–0.039; Table 4). Similarly, the lipidic model was then used in estimating 2‐year survival probabilities from MACE, which could merely differentiate patients with low risk in the multicentre cohort (log‐rank test, *P* = .049; Figure 5D). Our findings indicated that Cer(d18:1/24:1) contributed to CAD progression towards poor prognosis.

### 3.5 Lipid species associated with ACS

In the internal training cohort, univariate logistic regression analysis identified that 50 individual lipid species were significantly related to ACS (FDR < 0.05, Supporting information Table S8), of which 40 lipid species were still related to ACS (*P *< .05, Supporting information Table S8) after adjusting for confounding factors. Of note, eight lipids (LPC(16:1/0:0), LPC(18:1/0:0), LPC(18:2/0:0), LPC(18:3/0:0), LPC(20:2/0:0), LPC(20:3/0:0), LPC(22:0/0:0), and PC(18:2/20:4)) showed consistent relationships with the study by Meikle et al which revealed the significant plasma lipids associated with unstable CAD.^11^ Furthermore, pathway analysis revealed that glycerophospholipid, linoleic acid, and alpha‐linolenic acid metabolism were the top three disturbed pathways in patients with ACS (vs. those with stable CAD, Supporting information Figure S3B and Table S9).

In the external validation cohort, seven lipid metabolites showed statistical difference in univariate logistic analysis (*P* < .05), and four lipid species (LPC(18:3/0:0), LPE(0:0/24:6), LPC(22:0/0:0), and PC(18:2/18:2)) showed statistical difference in adjusted logistic analysis (*P* < .05, Supporting information Table S10) with ACS. Particularly, LPE(0:0/24:6) showed not only a negative correlation with ACS but also an independent protective biomarker for death risk in patients with CAD. These findings indicated that higher concentration of LPE(0:0/24:6) may decrease the risk of death by maintaining the plaque stability in patients with stable CAD.

### 3.6 Relationship between lipid species associated with clinical outcomes and LV remodeling

In the internal training cohort, among the 79 lipid species associated with clinical endpoints, 21 lipid species and four lipid species were correlated with LVEF and LVMI, respectively, after adjustment for covariates was performed (Supporting information Table S11). Four lipid species (HexCer(d18:1/22:0), HexCer(d18:1/26:0), HexCer(d18:1/26:1), and PE(34:1)) were significantly associated with LVEF and LVMI. Regarding the 11 lipid species that independently predicted the risks of clinical endpoints, Cer(d18:1/20:1) (estimate [SE], −1.07 [0.38]; *P* = 3.25E‐03) and Cer(d18:1/24:1) (estimate [SE], −1.19 [0.45]; *P* = 8.64E‐03) were negatively correlated with LVEF. In the external validation cohort, we found that Cer(d18:1/20:1) (estimate [SE], −0.27 [0.73]; *P* = 4.77E‐03) and CerP(d18:1/18:1) (estimate [SE], −2.06 [0.75]; *P* = 6.15E‐03) were significantly associated with LVEF (Supporting information Table S12). These findings suggested that ceramides may affect LV dysfunction before the occurrence of clinical endpoints.

## 4 DISCUSSION

This prospective work described a comprehensive lipidomic evaluation for the clinical endpoints of 1569 CAD patients’ prognoses and identified differences between ACS and stable CAD in the plasma lipids of two independent cohorts. We first illustrated that ceramides can considerably affect LV dysfunction before the occurrence of clinical endpoints. Moreover, the lipidic model consisting of independent lipid species and traditional risk factors shows considerably better predictive performance for 5‐year death risk than traditional model consisting only of traditional markers in the internal training cohort and yielded a successful 2‐year risk stratification in the external validation cohort. Lastly, the negative relationship between the structural characteristics and HR suggests that PC species enriched with polyunsaturated fatty acids may decrease death risk, whereas PE species enriched with less carbon atoms can increase death risk. These lipid species associated with clinical outcomes and LV malfunction may represent novel information about molecular targets and disease status independent of traditional plasma lipids.

### 4.1 Sphingolipid metabolism associated with clinical outcomes and LV function

We observed that ceramides were directly related to death and MACE risks in CAD patients, and our findings were accordant with those of previous studies.^13^, ^15^, ^16^, ^17^, ^19^ Ceramides are involved in plaque formation. The suppression of serine palmitoyltransferase, the rate‐limiting enzyme in denovo ceramides synthesis, prevents plaque development and enables the regression of preformed lesions in Apoe^–/–^ mice.^23^ Sphingosine‐1‐phosphate (S1P), the downstream metabolite of ceramides, is involved in sphingosine kinase‐S1P‐S1P receptor axis and angiogenesis regulation, because S1P1R, which is known for its important roles in angiogenesis, is present in endothelial cells at high levels. The knockout of S1P1R alone^24^ or the knockout of SK1 and SK2 simultaneously^25^ lead to embryonic lethality because of adverse vascular development. Endogenous ceramide participates in the transcytosis of oxidized LDL (oxLDL) through endothelial cells,^26^ regulation of monocyte adhesion to vessel walls, and succeeding LDL uptake increase.^27^ Hence, ceramides may affect the prognosis of patients with CAD by regulating plaque formation, angiogenesis, and lipid retention in vascular walls.

Ceramides are biomarkers of clinical outcomes and possibly cause CAD progression. We are the first to illustrate that ceramides may gradually lead to clinical endpoints by causing LV remodeling. Ceramides are significantly associated with LV malfunction.^18^ Very long‐chain ceramides could lead to mitochondrial damage, which in turn results in oxidative stress and in the death of cardiomyocytes.^28^ Experimental studies suggested that the cardiac‐specific increase in ceramides leads to cardiac dysfunction in animal models^29^, ^30^, ^31^ and that ceramide‐lowering interventions ameliorate atherosclerosis.^32^, ^33^ These findings indicate the potential of new intervention strategies in the modification of sphingolipid metabolism and attenuation of disease progression.

### 4.2 Dysregulation of glycerophospholipid metabolism involved in CAD progression

In this trial, glycerophospholipid metabolism was the most significant pathway in patients with higher risks of death and ACS. Different phosphatidylcholine (PC) and alkylphosphatidylcholine species showed diverse effects on death risk, which prompted our exploration of PC's biological activities. PC has pro‐ and anti‐inflammatory activities with a variety of oxidative modifications to polyunsaturated sn‐2 fatty acyl substituents.^34^ Some of these lipid species are implicated in the formation of oxLDL^35^ and atherosclerotic lesions.^36^ PC molecules include a lot of acyl chains that differ in length and double bond positions. The higher the content of double bonds in PC species is, the lower the death risk of CAD patients is, and this research is accordant with the results by Toledo et al.^37^ However, Stegemann et al^38^ indicated that PC composition and CVD risk have no clear relationship. Hence, individual lipid species and their biological activities in CAD progression need further research.

LPC was the lipid class with the most prominent differences between ACS and stable CAD. Seven of 16 lipid species showed characteristics consistent with those mentioned in the research of Meikle et al, which identified differences between unstable and stable CAD in terms of the plasma lipidome.^11^ These relationships may confirm that LPC maintains the stability of atherosclerosis plaque in patients with CAD, as a previous study suggested that LPC could exert protective effects by inhibiting macrophage cholesterol biosynthesis, decreasing cellular cholesterol accumulation, and showing antiatherogenesis effects.^39^

Interestingly, a similar association was observed with LPE(0:0/24:6), which is the independent lipid that protects against death risk, thereby suggesting that this lipid could decrease the risk of death by preventing the rupture of plaque in patients with stable CAD. Different observations were found with LPE(0:0/24:0) without unsaturated fatty acid and LPE(0:0/24:1) with monounsaturated fatty acid, which were positively associated with death risk. Hence, our results indicated that PE played different roles in the progression of CAD. Previously biochemical research revealed that PE is synthesized by the CDP‐ethanolamine and phosphatidylserine decarboxylase (Psd) pathway, and the latter was specifically located in the mitochondria.^40^ The CDP‐ethanolamine pathway generates a series of PEs abundant in high‐saturated fatty acids, whereas PEs with high unsaturation were produced by the Psd pathway.^41^ While, the functional diversity between the PEs generated through the Psd and CDP‐ethanolamine pathways is unclear. Thus, the functional mechanism of lipid species with different saturation degrees should be thinning in the CAD progression.

### 4.3 Development of the prognostic models of death and MACE

Our main objective was to develop robust prognostic models to predict clinical endpoints. Multiple epidemiological analyses suggested that HDLC and LDLC are the independent predictors of CVD.^8^, ^42^ A recent study on 151 217 patients with CAD indicated that elevated plasma HDLC concentration does not confer significant benefits to the alleviation of CVD.^43^ Our findings also indicated that LDLCs are not correlated with the risk of clinical endpoints in patients with CAD, as reported by a previous study.^19^ Hence, identifying novel lipid metabolites that can be applied to the risk prediction and stratification of death and MACE of CAD is necessary.

Our newly developed lipidic model containing 10 individual lipid spechies and two traditional risk factors remarkably enhance the predictive value of death risk compared with the traditional model and was successfully applied to the differentiation of multicentre patients with CAD with high‐death risk. However, the model used in predicting the risk of a complex event, such as MACE, showed a negligible improvement compared with the traditional model. This result suggested that individual lipid species are suitable for the risk stratification of a well‐defined clinical outcome, such as death, whereas complex events, such as MACE, may require a comprehensive model. Although the prediction model of death successfully stratified multicentre patients with high risk, the effectiveness of this model in populations of other regions or countries still needs further study.

### 4.4 Limitations

Our study has three limitations to consider. First, this study was based on Chinese populations, and the sample size of the multicentre validation cohort was relatively small. Second, the selection of covariates into the adjusted analyses was challenging because of the incomplete personal characteristics and inconsistency of the relationship between the demographic characteristics and clinical endpoints in both cohorts. Last, 667 individual lipid species were measured in the internal training cohort. Among these, only 309 lipid species were repeatedly detected in the multicentre cohort, resulting in the lack of five independent predictors in the predictive model of death risk. Nonetheless, the predictive model of death risk was successfully used to differentiate the multicentre patients with CAD.

## 5 CONCLUSIONS

Multiple lipid species independent of plasma cholesterol are powerful predictors of death risk in patients with CAD. Ceramides may indicate novel targets for mortality prevention and reduction. The possible underlying mechanism is the reversal of LV remodeling. The newly developed lipidic model is a powerful panel for death risk stratification in patients with CAD. The association between structural characteristics of long‐chain unsaturated fatty acids and death risk highlighted the need for mechanistic research, which would characterize the role of individual lipid species in disease pathogenesis.

## ETHICS APPROVAL AND CONSENT TO PARTICIPATE

The investigation conformed with the principles outlined in the Declaration of Helsinki. This study was approved by the Medical Ethical Review Committee of Guangdong Provincial People’s Hospital. Informed consent was obtained from all participants.

## CONSENT FOR PUBLICATION

Not applicable.

## CONFLICT OF INTEREST

The authors declare that they have no conflict of interest.

## AUTHOR CONTRIBUTIONS

Min Qin analysed the data. Min Qin, Qian Zhu, Weihua Lai, and Shilong Zhong wrote the original draft of the manuscript. Qilin Ma, Chen Liu, Xiaoping Chen, Yuelin Zhang, Zixian Wang, Hui Chen, Hong Yan, Heping Lei, Shuyao Zhang, Xuekui Dong, Hong Wang, Min Huang, Qizhou Lian, and Shilong Zhong contributed to the conception of the project, the design of the study, and the editing of the manuscript.

## AVAILABILITY OF DATA AND MATERIALS

The data that support the findings of this study are available from Shilong Zhong upon reasonable request

## Supporting information

Supporting information

## References

[1] Musunuru K , Kathiresan S . Genetics of common, complex coronary artery disease. Cell. 2019;177:132‐145. 30901535 10.1016/j.cell.2019.02.015

[2] Benjamin EJ , Muntner P , Alonso A , et al. Heart disease and strokestatistics‐2019 update: a report from the American Heart Association. Circulation 2019;139:e56‐e528. 30700139 10.1161/CIR.0000000000000659

[3] Zhou M , Wang H , Zeng X , et al. Mortality, morbidity, and risk factors in China and its provinces, 1990–2017: a systematic analysis for the Global Burden of Disease Study 2017. Lancet. 2019;394:1145‐1158. 31248666 10.1016/S0140-6736(19)30427-1 PMC6891889

[4] Lindholm D , Lindback J , Armstrong PW , et al. Biomarker‐based risk model to predict cardiovascular mortality in patients with stable coronary disease. J Am Coll Cardiol. 2017;70:813‐826. 28797349 10.1016/j.jacc.2017.06.030

[5] Rapsomaniki E , Shah A , Perel P , et al. Prognostic models for stable coronary artery disease based on electronic health record cohort of 102 023 patients. Eur Heart J. 2014;35:844‐852. 24353280 10.1093/eurheartj/eht533 PMC3971383

[6] Savolainen MJ . Epidemiology: disease associations and modulators of HDL‐related biomarkers. Handb Exp Pharmacol. 2015;224:259‐283. 25522991 10.1007/978-3-319-09665-0_7

[7] Lin JS , Evans CV , Johnson E , et al. Nontraditional risk factors in cardiovascular disease risk assessment: updated evidence report and systematic review for the US preventive services taskforce. JAMA. 2018;320:281‐297. 29998301 10.1001/jama.2018.4242 PMC13564044

[8] Prospective Studies C , Lewington S , Whitlock G , et al. Blood cholesterol and vascular mortality by age, sex, and blood pressure: a meta‐analysis of individual data from 61 prospective studies with 55,000 vascular deaths. Lancet. 2007;370:1829‐1839. 18061058 10.1016/S0140-6736(07)61778-4

[9] Cheng PC , Hsu SR , Li JC , et al. Plasma low‐density lipoprotein cholesterol correlates with heartfunction in individuals with type 2 diabetes mellitus: a cross‐sectional study. Front Endocrinol (Lausanne). 2019;10:234. 31031709 10.3389/fendo.2019.00234 PMC6470411

[10] Mundra PA , Shaw JE , Meikle PJ . Lipidomic analyses in epidemiology. Int J Epidemiol. 2016;45:1329‐1338. 27286762 10.1093/ije/dyw112

[11] Meikle PJ , Wong G , Tsorotes D , et al. Plasma lipidomic analysis of stable and unstable coronary artery disease. Arterioscler Thromb Vasc Biol. 2011;31:2723‐2732. 21903946 10.1161/ATVBAHA.111.234096

[12] Mundra PA , Barlow CK , Nestel PJ , et al. Large‐scale plasma lipidomic profiling identifies lipids that predict cardiovascular events in secondary prevention. JCI Insight. 2018;3:e121326. 10.1172/jci.insight.121326 PMC6171797 30185661

[13] Laaksonen R , Ekroos K , Sysi‐Aho M , et al. Plasma ceramides predict cardiovascular death in patients with stable coronary artery disease and acute coronary syndromes beyond LDL‐cholesterol. Eur Heart J. 2016;37:1967‐1976. 27125947 10.1093/eurheartj/ehw148 PMC4929378

[14] Poss AM , Maschek JA , Cox JE , et al. Machine learning reveals serum sphingolipids as cholesterol‐independent biomarkers of coronary artery disease. J Clin Invest. 2020;130:1363‐1376. 31743112 10.1172/JCI131838 PMC7269567

[15] Havulinna AS , Sysi‐Aho M , Hilvo M , et al. Circulating ceramides predict cardiovascular outcomes in the population‐based FINRISK 2002 cohort. Arterioscler Thromb Vasc Biol. 2016;36:2424‐2430. 27765765 10.1161/ATVBAHA.116.307497

[16] Kauhanen D , Sysi‐Aho M , Koistinen KM , et al. Development and validation of a high‐throughput LC‐MS/MS assay for routine measurement of molecular ceramides. Anal Bioanal Chem. 2016;408:3475‐3483. 26922344 10.1007/s00216-016-9425-z

[17] Anroedh S , Hilvo M , Akkerhuis KM , et al. Plasma concentrations of molecular lipid species predict long‐term clinical outcome in coronary artery disease patients. J Lipid Res. 2018;59:1729‐1737. 29858423 10.1194/jlr.P081281 PMC6121931

[18] Nwabuo CC , Duncan M , Xanthakis V , et al. Association of circulating ceramides with cardiac structure and function in the community: the Framingham heart study. J Am Heart Assoc. 2019;8:e013050. 31549564 10.1161/JAHA.119.013050 PMC6806035

[19] Alshehry ZH , Mundra PA , Barlow CK , et al. Plasma lipidomic profiles improve on traditional risk factors for the prediction of cardiovascular events in type 2 diabetes mellitus. Circulation. 2016;134:1637‐1650. 27756783 10.1161/CIRCULATIONAHA.116.023233

[20] Wu D , Shu T , Yang X , et al. Plasma metabolomic and lipidomic alterations associated with COVID‐19. Natl Sci Rev. 2020;7:1157‐1168. 10.1093/nsr/nwaa086 PMC7197563 34676128

[21] Mohr FW , Morice MC , Kappetein AP , et al. Coronary artery bypass graft surgery versus percutaneous coronary intervention in patients with three‐vessel disease and left main coronary disease: 5‐year follow‐up of the randomised, clinical SYNTAX trial. Lancet. 2013;381:629‐638. 23439102 10.1016/S0140-6736(13)60141-5

[22] Serruys PW , Morice MC , Kappetein AP , et al. Percutaneous coronary intervention versus coronary‐artery bypass grafting for severe coronary artery disease. N Engl J Med. 2009;360:961‐972. 19228612 10.1056/NEJMoa0804626

[23] Park TS , Panek RL , Mueller SB , et al. Inhibition of sphingomyelin synthesis reduces atherogenesis in apolipoprotein E‐knockout mice. Circulation. 2004;110:3465‐3471. 15545514 10.1161/01.CIR.0000148370.60535.22

[24] Liu Y , Wada R , Yamashita T , et al. Edg‐1, the G protein‐coupled receptor for sphingosine‐1‐phosphate, is essential for vascular maturation. J Clin Invest. 2000;106:951‐961. 11032855 10.1172/JCI10905 PMC314347

[25] Mizugishi K , Yamashita T , Olivera A , et al. Essential role for sphingosine kinases in neural and vascular development. Mol Cell Biol. 2005;25:11113‐11121. 16314531 10.1128/MCB.25.24.11113-11121.2005 PMC1316977

[26] Li W , Yang X , Xing S , et al. Endogenous ceramide contributes to the transcytosis of oxLDL across endothelial cells and promotes its subendothelial retention in vascular wall. Oxid Med Cell Longev. 2014;2014:823071. 24817993 10.1155/2014/823071 PMC4003761

[27] Gao D , Pararasa C , Dunston CR , et al. Palmitate promotes monocyte atherogenicity via de novo ceramide synthesis. Free Radic Biol Med. 2012;53:796‐806. 22640955 10.1016/j.freeradbiomed.2012.05.026

[28] Law BA , Liao X , Moore KS , et al. Lipotoxic very‐long‐chain ceramides cause mitochondrial dysfunction, oxidative stress, and cell death in cardiomyocytes. FASEB J. 2018;32:1403‐1416. 29127192 10.1096/fj.201700300R PMC5892719

[29] Son NH , Park TS , Yamashita H , et al. Cardiomyocyte expression of PPARgamma leads to cardiac dysfunction in mice. J Clin Invest. 2007;117:2791‐2801. 17823655 10.1172/JCI30335 PMC1964508

[30] Chiu HC , Kovacs A , Ford DA , et al. A novel mouse model of lipotoxic cardiomyopathy. J Clin Invest. 2001;107:813‐822. 11285300 10.1172/JCI10947 PMC199569

[31] Yagyu H , Chen G , Yokoyama M , et al. Lipoprotein lipase (LpL) on the surface of cardiomyocytes increases lipid uptake and produces a cardiomyopathy. J Clin Invest. 2003;111:419‐426. 12569168 10.1172/JCI16751 PMC151861

[32] Glaros EN , Kim WS , Quinn CM , et al. Myriocin slows the progression of established atherosclerotic lesions in apolipoprotein E gene knockout mice. J Lipid Res. 2008;49:324‐331. 17978313 10.1194/jlr.M700261-JLR200

[33] Glaros EN , Kim WS , Wu BJ , et al. Inhibition of atherosclerosis by the serine palmitoyl transferase inhibitor myriocin is associated with reduced plasma glycosphingolipid concentration. Biochem Pharmacol. 2007;73:1340‐1346. 17239824 10.1016/j.bcp.2006.12.023

[34] Ke Y , Zebda N , Oskolkova O , et al. Anti‐inflammatory effects of OxPAPC involve endothelial cell‐mediated generation of LXA4. Circ Res. 2017;121:244‐257. 28522438 10.1161/CIRCRESAHA.116.310308 PMC5886749

[35] Harrison KA , Davies SS , Marathe GK , et al. Analysis of oxidized glycerophosphocholine lipids using electrospray ionization mass spectrometry and microderivatization techniques. J Mass Spectrom. 2000;35:224‐236. 10679985 10.1002/(SICI)1096-9888(200002)35:2<224::AID-JMS933>3.0.CO;2-G

[36] Podrez EA , Poliakov E , Shen Z , et al. A novel family of atherogenic oxidized phospholipids promotes macrophage foam cell formation via the scavenger receptor CD36 and is enriched in atherosclerotic lesions. J Biol Chem. 2002;277:38517‐38523. 12145296 10.1074/jbc.M205924200

[37] Toledo E , Wang DD , Ruiz‐Canela M , et al. Plasma lipidomic profiles and cardiovascular events in a randomized intervention trial with the Mediterranean diet. Am J Clin Nutr. 2017;106:973‐983. 28814398 10.3945/ajcn.116.151159 PMC5611779

[38] Stegemann C , Pechlaner R , Willeit P , et al. Lipidomics profiling and risk of cardiovascular disease in the prospective population‐based Bruneck study. Circulation. 2014;129:1821‐1831. 24622385 10.1161/CIRCULATIONAHA.113.002500

[39] Rozenberg O , Shih DM , Aviram M . Human serum paraoxonase 1 decreases macrophage cholesterol biosynthesis:possible role for its phospholipase‐A2‐like activity and lysophosphatidylcholine formation. Arterioscler Thromb Vasc Biol. 2003;23:461‐467. 12615663 10.1161/01.ATV.0000060462.35946.B3

[40] Birner R , Bürgermeister M , Schneiter R , et al. Roles of phosphatidylethanolamine and of its several biosynthetic pathways in *Saccharomyces cerevisiae* . Mol Biol Cell. 2001;12:997‐1007. 11294902 10.1091/mbc.12.4.997 PMC32282

[41] Calzada E , Onguka O , Claypool SM . Phosphatidylethanolamine metabolism in health and disease. Int Rev Cell Mol Biol. 2016;321:29‐88. 26811286 10.1016/bs.ircmb.2015.10.001 PMC4778737

[42] Emerging Risk Factors C , Di Angelantonio E , Sarwar N , et al. Major lipids, apolipoproteins, and risk of vascular disease. JAMA. 2009;302:1993‐2000. 19903920 10.1001/jama.2009.1619 PMC3284229

[43] Millwood IY , Bennett DA , Holmes MV , et al. Association of CETP gene variants with risk for vascular and nonvascular diseases among Chinese adults. JAMA Cardiol. 2018;3:34‐43. 29141072 10.1001/jamacardio.2017.4177 PMC5833522

