# Supplementary material for: Insights into the prognosis of lipidomic dysregulation for death risk in patients with coronary artery disease
Source: Clin Transl Med. 2020 Sep 28;10(5):e189. doi: 10.1002/ctm2.189 (PMC7522592; doi:10.1002/ctm2.189)
Supplement: Supplementary file 1 — Supporting information [file CTM2-10-e189-s001.docx]

**Supporting Information**

**Insights into the prognosis of lipidomic dysregulation for death risk in patients with coronary artery disease**

Min Qin, ^a, b, c #^ Qian Zhu, ^a, b, c #^ Weihua Lai, ^a #^ Qilin Ma, ^d^ Chen Liu, ^e^ Xiaoping Chen, ^d^ Yuelin Zhang, ^f^ Zixian Wang, ^a, b, g^ Hui Chen, ^a, b, c^ Hong Yan, ^b, c^ Heping Lei, ^b^ Shuyao Zhang, ^h^ Xuekui Dong, ^i^ Hong Wang, ^i^ Min Huang, ^j^ Qizhou Lian, ^k^ Shilong Zhong ^a, b, c, g *^

^a^Department of Pharmacy, Guangdong Provincial People's Hospital, Guangdong Academy of Medical Sciences, Guangzhou, Guangdong 510080, China.

^b^Guangdong Provincial Key Laboratory of Coronary Heart Disease Prevention, Guangdong Cardiovascular Institute, Guangdong Provincial People's Hospital, Guangdong Academy of Medical Sciences, Guangzhou, Guangdong 510080, China.

^c^School of Medicine, South China University of Technology, Guangzhou, Guangdong 510080, China.

^d^Department of Clinical Pharmacology, Xiangya Hospital, Central South University, Changsha, Hunan 410008, China.

^e^Department of Cardiology, The First Affiliated Hospital, Sun Yat-sen University, Guangzhou, Guangdong 510080, China.

^f^Department of Emergency Medicine, Department of Emergency and Critical Care Medicine, Guangdong Provincial People's Hospital, Guangdong Academy of Medical Sciences, Guangzhou, Guangdong 510080, China.

^g^School of Biology and Biological Engineering, South China University of Technology, Guangzhou 510006, China.

^h^Guangzhou Red Cross Hospital affiliated to Ji-Nan University Medical College, Guangzhou Guangdong 510000, China.

^i^Wuhan Metware Biotechnology Co., Ltd., Wuhan, Hubei 430000, China.

^j^Institute of Clinical Pharmacology, School of Pharmaceutical Sciences, Sun Yat-Sen University, Guangzhou, Guangdong 510006, China.

^k^Department of Medicine, The University of Hong Kong, Hong Kong SAR, P.R. China.

**Address for correspondence**

^*^Shilong Zhong, Ph.D. Professor

Department of Pharmacy,

Guangdong Provincial Key Laboratory of Coronary Heart Disease Prevention,

Guangdong Provincial People's Hospital,

Guangdong Academy of Medical Sciences.

106 Zhongshan Road, Weilun Bldg.1112,

Guangzhou 510080, P. R. China

Tel: +8620-83827812 - 60298

Email: [gdph_zhongsl@gd.gov.cn](mailto:gdph_zhongsl@gd.gov.cn)

**Running Head:** Lipidomic dysregulation on the death risk of CAD

^#^Min Qin, Qian Zhu and Weihua Lai contributed equally to the study and are considered co-first authors.

**Figure S1.** ESI full scan mass spectrum ion pairs of 11 independent lipid species

**Figure S2.** 10-fold cross-validation for tuning parameter selection in the lasso Cox model

**Figure S3.** Pathway analysis of lipid species associated with death and ACS in CAD patients

**Figure S4.** Bubble charts of PC and PE replicated in external validation cohort

**Table S1.** Conditions for tandem mass spectrometry analysis of lipid species

**Table S2.** Baseline characteristics and effects on LVEF and LVMI risks in internal training and external validation cohorts

**Table S3.** Relationship between lipid species and death in univariate and adjusted Cox analyses of the internal training cohort

**Table S4.** Relationship between lipid species and MACE in univariate and adjusted Cox analyses of the internal training cohort

**Table S5.** Relationship between lipid species and death in univariate and adjusted Cox analyses of external validation cohort

**Table S6.** Disturbed metabolic pathways in CAD patients with higher risks from death

**Table S7.** Multivariable Cox proportional hazard model for MACE

**Table S8.** Lipid species associated with ACS (vs. stable CAD) in the internal training cohort

**Table S9.** Disturbed metabolic pathways in patients with ACS vs. stable CAD

**Table S10.** Lipid species associated with ACS (vs. stable CAD) in the external validation cohort

**Table S11.** Association of the prognostic lipid species with LVEF and LVMI in the internal training cohort

**Table S12.** Association of the prognostic lipid species with LVEF and LVMI in the external validation cohort

**Supplementary Methods**

**Study populations**

Patients in the internal training cohort were sequentially and prospectively enrolled in Guangdong Provincial People’s Hospital between January 2010 and December 2013. They were followed up for the primary endpoint (all-cause death) and the secondary endpoint (major adverse cardiovascular event, MACE) from June 2010 to April 2017 for up to 5 years. Patients in the external validation cohort were sequentially enrolled from Guangdong Provincial People’s Hospital (n = 347), Xiangya Hospital of Centre-south University (n = 178), and First Affiliated Hospital of Sun Yat-sen University (n = 33) from September 2017 to October 2018. These patients were followed up until December 2019 for a median of 1.13 years.

The primary endpoint of interest is the all-cause death, and the secondary endpoint of interest is MACE. MACE was defined as the occurrence of death, nonfatal myocardial infarctions, coronary revascularisation and cerebral infraction. All participants were followed up prospectively for the study endpoints based on inpatient and outpatient hospital visits and telephone contacts with the patients or their families. At each follow-up assessment conducted every 6 months, the participants were asked about the occurrence of new adverse cardiovascular events. Baseline information, including demographics, medical history, biochemical measurements and medication, was obtained from the hospital information database. Coronary angiography (CAG) was performed. The SYNTAX score was calculated to assess the severity of CAD patients. Echocardiography was applied to determine the LV function and structure.

**Assessment of CAD severity by CAG**

CAG was performed using standard technique, and images of CAG were obtained with Syngo Dynamics cardiovascular imaging software (Siemens Medical Solutions, USA, Inc., Malvern, Pennsylvania). All angiograms were assessed by two expert cardiologists who were blinded to the metabolomics data. The SYNTAX score was used to determine CAD complexity and severity [[1](#_ENREF_1)]. The SYNTAX score characterised the anatomy of coronary vasculature with respect to the lesions number, lesion location, the occurrence of total occlusions, bridging collaterals, bi/trifurcations, aorto-ostial, tortuosity, lesion length, calcification, thrombus and diffuse disease/small vessels. The SYNTAX score was calculated for each patient by using the online SYNTAX score calculator version 2.11.

**Assessment of LV function and structure by echocardiography**

Echocardiography was performed by two experienced cardiologists by using a Philips iE33 system (Philips Medical Systems, Bothell, WA, USA) to assess the LV structure and function in accordance with the European and American Guidelines of Echocardiography [[2](#_ENREF_2)]. LV end-diastolic dimension, interventricular septal thickness and posterior wall thickness were measured at end diastole. LV mass (LVM) was calculated based on the Devereux formula. Body surface area (BSA) was calculated using the Stevenson formula. Thereafter, the left ventricular mass index (LVMI) was obtained by dividing LVM by BSA. LV ejection fraction (LVEF) was evaluated by the modified Simpson rule.

**Widely targeted lipidomics profiling**

In the internal training and external validation sets, the widely targeted lipidomic profiling was performed using ultra-performance liquid chromatography mass spectrometry (UPLC-MS/MS) system (UPLC, Shim-pack UFLC SHIMADZU CBM30A; MS, Applied Biosystems SCIEX 6500+ QTRAP) at Wuhan Metware Biotechnology. Totally, 667 plasma endogenous lipid species consisting of 14 lipid classes/subclasses and 687 lipid species containing 20 lipid classes/subclasses were annotated in the internal training and external validation cohorts, respectively. Approximately 309 identical lipid species were detected in both cohorts. These lipid species mainly include monoglyceride (MG), cholesteryl esters (CE), diacylglycerol (DG), triacylglycerol (TG), phosphatidic acids (PA), phosphatidylcholines (PC), phosphatidylglycerol (PG), phosphatidylserines (PS), phosphatidylethanolamines (PE), lysophosphatidic acids (LPA), lysophosphatidylcholine (LPC), lysophosphatidylethanolamine (LPE), hemolytic serine (LPS) and ceramides (Cer). The ESI full scan mass spectra ion pairs and conditions for tandem mass spectrometry analysis of the lipid species are shown in *Figure S1* and *Table S1*, respectively.

Lipid species were extracted from the plasma of CAD patients. Firstly, the sample was thawed on ice, whirled for 10 s and centrifuged with 3000 r/min at 4 ℃ for 5 min. Secondly, 50 μL of plasma and 1 mL of lipid extraction reagent were pooled into the corresponding numbered centrifuge tube. The mixture was vortexed for 2 min, added with 500 μL of deionised water, vortexed for 1 min and centrifuged with 12,000 r/min at 4 ℃ for 10 min. Thirdly, 500 μL of supernatant was absorbed into the numbered centrifuge tube and concentrated after centrifugation. Lastly, the powder was dissolved with 100 μL of mobile phase B (comprising 10% acetonitrile, 90% isopropanol, 0.04% acetic acid, and 5 mmol/L ammonium formate), and the dissolving solution was then used for UPLC-MS/MS analysis.

The calibration and quality control (QC) samples were prepared with the mixed plasma of subjects prior to sample analysis. Every 10 samples to be analysed were separated by one QC sample for the duration of the detection to monitor repeatability during the analysis. The repeatability of lipid extraction and detection was judged by the overlapping analysis of total ion flow diagrams between different QC samples. The high overlaps of the total ion flow, that is, the retention time and peak strength are consistent, indicates that the signal stability of the mass spectrum is good at different times.

The separation was performed in a Thermo C30 column (2.6 µM, 2.1 mm × 100 mm). A UPLC column (Thermo C30, 2.6 μm, 2.1 mm*100 mm) was used at a column temperature of 45〬C. The mobile phase was composed of acetonitrile/water (60/40, V/V) containing 0.04% acetic acid, 5 mmol/L ammonium formate (A) and acetonitrile/isopropanol (10/90, V/V) containing 0.04% acetic acid and 5 mmol/L ammonium formate (B). The gradient program initiated from 20% B to 50% B at 3.0 min, to 65% B at 5 min, to 75% B at 9 min and to 90% B at 15.5 min with a flow rate at 0.35 mL/min. The injection volume was set at 2 μL. The effluent was alternatively connected to an ESI-QTRAP-MS.

The LIT and triple quadrupole (QQQ) scans were acquired on a QTRAP-MS (QTRAP® 6500+ LC-MS/MS System) equipped with an ESI Turbo Ion-Spray interface operating in positive and negative ion modes and controlled by Analyst 1.6.3 software (Sciex). The ESI source operation parameters were as follows: ion source, turbo spray; source temperature of 550 °C and ion spray voltage of 5500 V. The ion source gas I, gas II and curtain gas were set at 55, 60, and 25 psi, respectively. The collision gas was medium. Instrument tuning and mass calibration were performed with 10 and 100 μmol/L polypropylene glycol solutions in QQQ and LIT modes, respectively. The QQQ scans were acquired as MRM experiments with collision gas (nitrogen) set to 5 psi. The DP and CE for individual MRM transitions were conducted with further DP and CE optimisation. A specific set of MRM transitions were monitored for each period according to the metabolites eluted within this period.

Qualitative analysis of the MS and MS/MS mass spectrometric data was performed on the basis of the home-made database Metware database (MWDB) and the public database of metabolite information. The lipid metabolite structural analysis mainly referred to MassBank, KNAPSAcK, HMDB, Lipidmaps and METLIN database. Analyst 1.6.3 software (AB Sciex) was used to process the raw mass spectrometry data.

**Statistical analysis**

For baseline characteristics, the counts (percentages) and mean (SD) were used to represent the categorical and continuous variables, respectively. For the lipidic data, raw signals with >50% missing values in the quality control (QC) samples, i.e., those with zero ion intensity, were removed. Samples for the undetected lipid species were imputed with the minimum detected level for the lipid species. To reduce the bias from the batch effect, Quality Control–Robust Loess Signal Correction (QC-RLSC) algorithm was used to correct and integrate eight batches in the internal training and external validation cohorts [[3](#_ENREF_3)]. QC-RLSC is an effective way to normalise the lipid features to the QC samples within an analytical block. Each block of the lipidomic data was then scaled through pareto scaling with procedures of mean cantering and scaling to the square root of the standard deviation.

The prognostic model of death was constructed by least absolute shrinkage and selection operator (Lasso) Cox regression analysis. Lasso is a method that is frequently used for the analysis of the regression of high-dimensional predictors. Variable selection and regularisation are performed to enhance the prediction accuracy and interpretability of the statistical model [[4](#_ENREF_4), [5](#_ENREF_5)]. The method involves the use of an L1 penalty to shrink some regression coefficients to exactly zero. The penalty parameter λ, which is known as the tuning parameter, controls the amount of shrinkage. The larger the λ value is, the fewer the selected predictors are. Lasso has been extensively applied to the Cox proportional hazard regression model for survival analysis with high-dimensional data. This approach can also be used for the optimal selection of markers in high-dimensional data with strong prognostic value and low correlation among one another to prevent overfitting. A 10-fold cross-validation was used to determine the optimal values of λ. This procedure was repeated 200 times. The markers left in the model required selection for over 180 times out of 200 repetitions. The final regression coefficient of each selected feature was calculated by using the average of all coefficients.

Time-dependent receiver-operating characteristic (ROC) analysis was performed by using the “timeROC” package in R to assess the prognosis of the proportional hazards models for death and MACE. A large area under the curve (AUC) value resulted in great predictive discrimination of the model. The external validation of prognostic models was performed in the multicentre cohort based on the calculation of individual hazard estimates. The hazard stratification was presented by Kaplan–Meier curves between among low (<Q1), middle (≥Ql and ≤Q3) and high (>Q3) hazard estimates.

**References**

1. Sianos G, Morel MA, Kappetein AP, et al. The SYNTAX Score: an angiographic tool grading the complexity of coronary artery disease. EuroIntervention. 2005; 1: 219-27.

2. Lang RM, Bierig M, Devereux RB, et al. Recommendations for chamber quantification. Eur J Echocardiogr. 2006; 7: 79-108.

3. Luan H, Ji F, Chen Y, et al. statTarget: A streamlined tool for signal drift correction and interpretations of quantitative mass spectrometry-based omics data. Anal Chim Acta. 2018; 1036: 66-72.

4. Tibshirani RJJotRSS. Regression Shrinkage and Selection via the Lasso. 1996; 58: 267-88.

5. Tibshirani R, . %J Statistics in Medicine. The lasso method for variable selection in the Cox model. 1997; 16: 385-95.


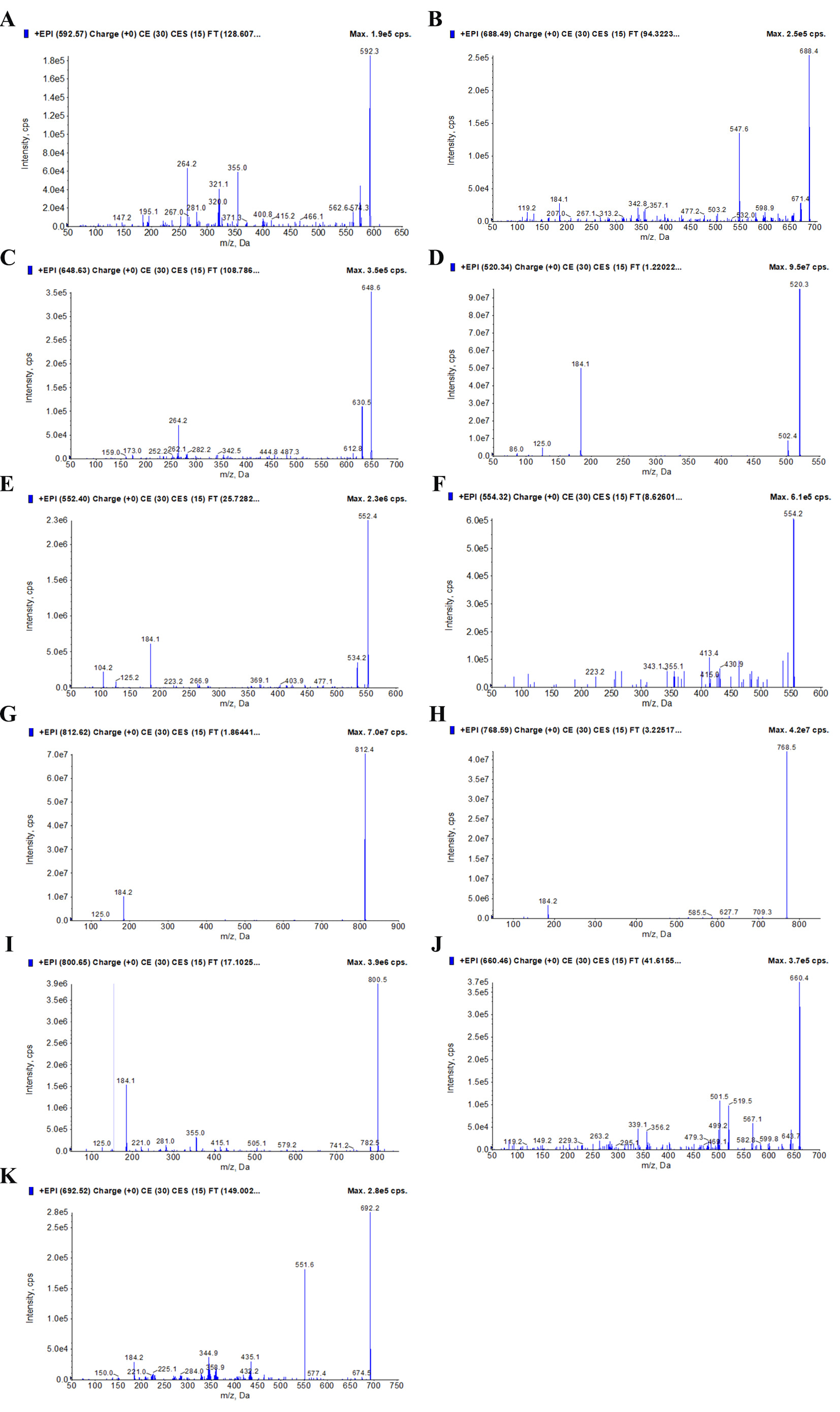


**Figure S1. ESI full scan mass spectrum ion pairs of 11 independent lipid species.** Cer(d18:1/20:1) **(A)**, PE(32:2) **(B)**, Cer(d18:1/24:1) **(C)**, LPC(18:2/0:0) **(D)**, LPC(20:0/0:0) **(E)**, LPE(0:0/24:6) **(F)**, PC(16:1/22:2) **(G)**, PC(O-36:4) **(H)**, PC(O-38:2) **(I)**, PE(30:2) **(J)**, and PE(32:0) **(K)**.


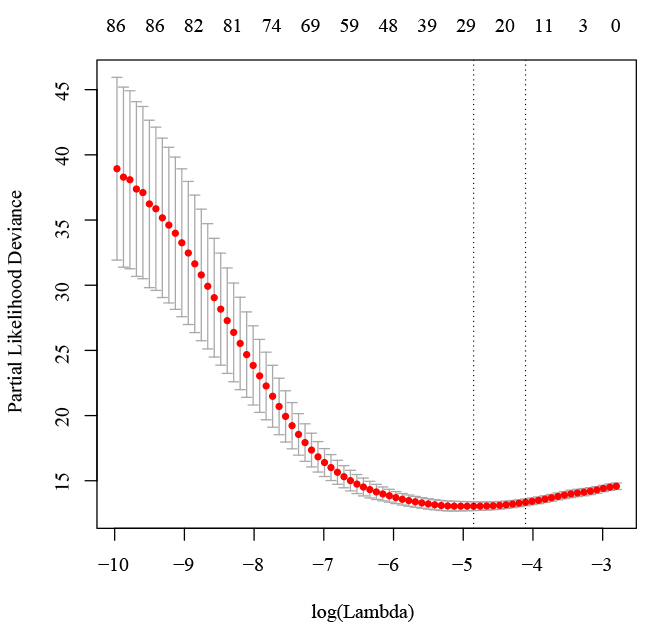


**Figure S2. 10-fold cross-validation for tuning parameter selection in the lasso Cox model.** Solid vertical lines represent partial likelihood deviance ± standard error (SE). The dotted vertical lines were drawn at the optimal values by minimum and 1-SE criteria. We plotted the partial likelihood deviance versus log (λ), where λ is the tuning parameter. Herein, λ = 0.016 with log (λ) = −4.106 was chosen by 10-fold cross-validation via 1-SE criteria.


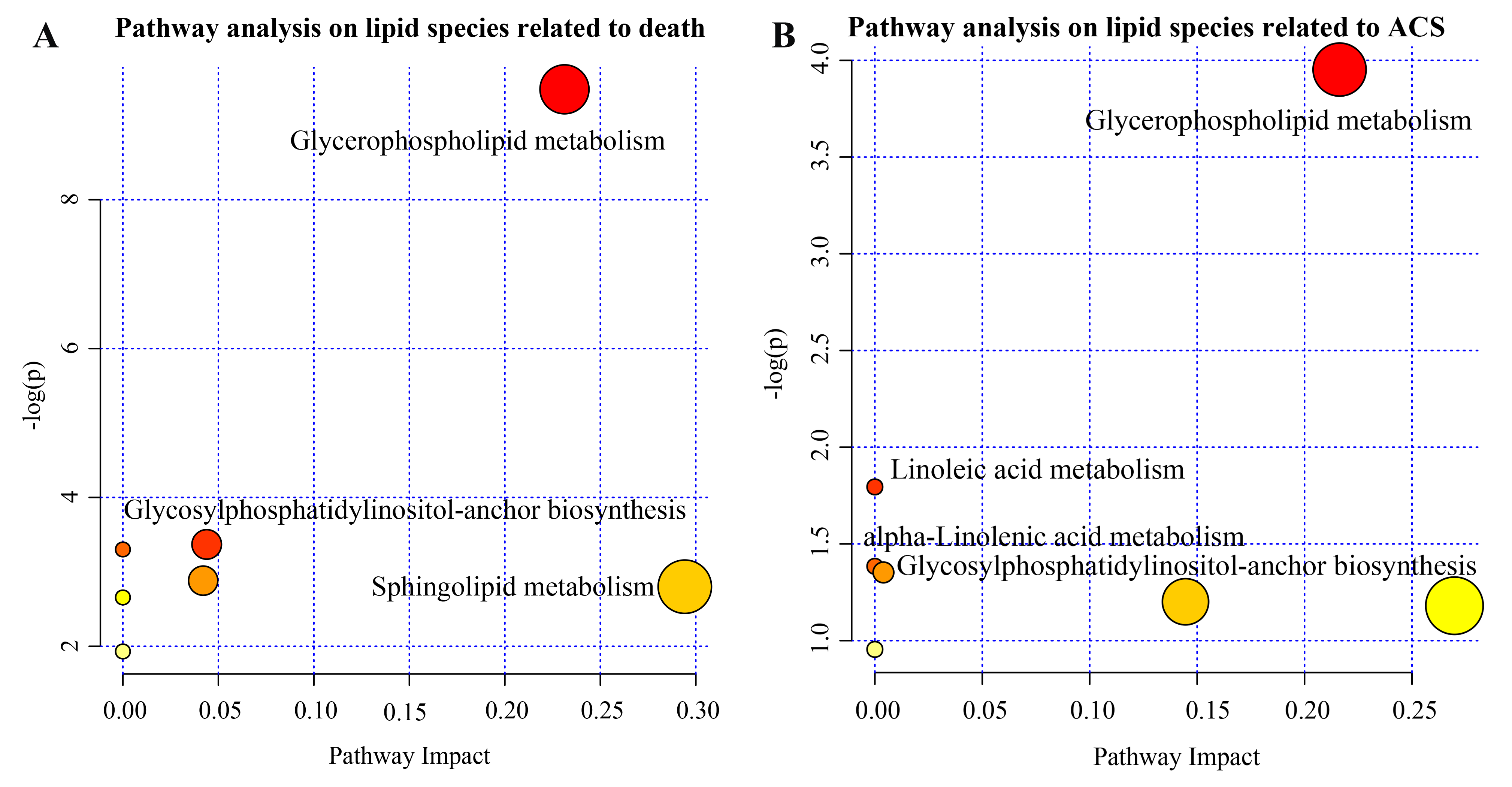


**Figure S3. Pathway analysis of lipid species associated with death (A) and ACS (B) in CAD patients.** Biochemical pathway analysis revealed the top 3 disturbed metabolic pathways in patients with higher risks of death (**A**) and ACS (**B**). The size and color of each circle were based on pathway impact value and *P*-value, respectively.

**
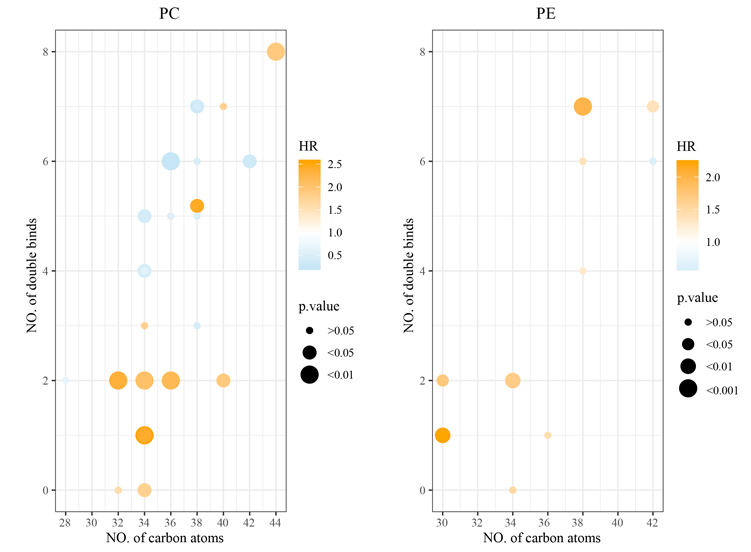
**

**Figure S4. Bubble charts of PC and PE replicated in external validation cohort.** HRs were calculated from adjusted Cox analysis with adjustments for age, AST, creatine kinase (CK) and eGFR levels.

**Table S1. Conditions for tandem mass spectrometry analysis of lipid species**

| **Lipid Class** | **Parent Ion** | **Fragmentation** | **Number of features** | **Internal Standard** | **Internal standard (pmol)** | **Collision Energy (V)** |
| --- | --- | --- | --- | --- | --- | --- |
| Dihydroceramide (Cer(d18:0)) | [M+H]^+^ | NL, 18 Da | 4 | Cer(d18:1/17:0) | 10 | 40 |
| Ceramide (Cer(d18:1)) | [M+H]^+^ | PI, m/z 264.3 | 6 | Cer(d18:1/17:0) | 10 | 40 |
| Ceramide (Cer(t18:0)) | [M+H]^+^ | PI, m/z 264.3 | 4 | Cer(d18:1/17:0) | 10 | 40 |
| Ceramide (Cer(m18:1)) | [M+H]^+^ | PI, m/z 264.3 | 10 | Cer(d18:1/17:0) | 10 | 40 |
| Ceramide-1-Phosphate (CerP) | [M+H]^+^ | PI, m/z 264.3 | 10 | Cer(d18:1/17:0) | 10 | 40 |
| Phosphatidylcholine (PC) | [M+H]^+^ | PI, m/z 184.1 | 86 | PC(13:0/13:0) | 10 | 30 |
| Alkylphosphatidylcholine (PC(O)) | [M+H]^+^ | PI, m/z 184.1 | 47 | PC(13:0/13:0) | 10 | 30 |
| Lysophosphatidylcholine (LPC) | [M+H]^+^ | PI, m/z 184.1 | 19 | LPC(12:0) | 10 | 30 |
| Lysoalkylphosphatidylcholine (LPC(O)) | [M+H]^+^ | PI, m/z 184.1 | 14 | LPC(12:0) | 10 | 30 |
| Phosphatidylethanolamine (PE) | [M+H]^+^ | NL, 141 Da | 40 | PE(12:0/12:0) | 10 | 30 |
| Alkenylphosphatidylethanolamine (PE(P)) | [M+H]^+^ | NL, 141 Da | 35 | PE(12:0/12:0) | 10 | 30 |
| Lysophosphatidylethanolamine (LPE) | [M+H]^+^ | NL, 141 Da | 19 | LPE(14:0) | 10 | 30 |
| AlkenylLysophosphatidylethanolamine (LPE(P)) | [M+H]^+^ | NL, 141 Da | 2 | LPE(14:0) | 10 | 30 |
| Phosphatidylglycerol (PG) | [M+H]^+^ | acyl specific | 1 | PG(12:0/12:0) | 10 | 30 |
| Cholesterol ester (CE) | [M+NH_4_]^+^ | PI, m/z 369.3 | 8 | CE(17:0) | 100 | 20 |
| Monoacylglycerol (MG) | [M+NH_4_]^+^ | NL, 91 Da | 4 | MG(17:0) | 10 | 20 |
| Diacylglycerol (DG) | [M+NH_4_]^+^ | NL, NH_3_ + fatty acid | 40 | DG(15:0/15:0) | 10 | 30 |
| Triacylglycerol (TG) | [M+NH_4_]^+^ | NL, NH_3_ + fatty acid | 296 | TG(17:0/17:0/17:0) | 10 | 30 |

| **Table S2 Baseline characteristics and the effects on LVEF and LVMI risks in the internal training cohort and external validation cohort** | | | | | | | | | | |
| --- | --- | --- | --- | --- | --- | --- | --- | --- | --- | --- |
|  | **Internal training cohort** | | | | | **External validation cohort** | | | | |
| **Characteristics** | **Value N (%) or mean ± SD** | **LVEF** | | **LVMI** | | **Value N (%) or mean ± SD** | **LVEF** | | **LVMI** | |
|  |  | **Estimate ± SE** | **P Value** | **Estimate ± SE** | **P Value** |  | **Estimate ± SE** | **P Value** | **Estimate ± SE** | **P Value** |
| **Demographic data** |  |  |  |  |  |  |  |  |  |  |
| age | 63.01 ±10.07 | -0.02±0.04 | 5.90E-01 | 0.16±0.14 | 2.58E-01 | 62.29±10.20 | -0.09±0.05 | 1.01E-01 | 0.28±0.21 | 1.83E-01 |
| SEX（male） | 805(79.62) | -4.10±1.01 | 5.85E-05 | 10.64±3.45 | 2.15E-03 | 414(74.19) | -2.43±1.23 | 4.86E-02 | 4.43±5.08 | 3.83E-01 |
| BMI,kg/m² | 24.27 ±4.82 | 0.10±0.08 | 2.41E-01 | -0.01±0.27 | 9.81E-01 | 24.04±3.35 | 0.18±0.19 | 3.70E-01 | -1.39±0.61 | 2.28E-02 |
| Comorbidities |  |  |  |  |  |  |  |  |  |  |
| arrhythmia | 88(8.72) | -3.58±1.43 | 1.26E-02 | 18.52±4.82 | 1.35E-04 | 51(9.14) | 0.27±1.82 | 8.83E-01 | 1.24±7.05 | 8.60E-01 |
| Diabetes | 277(27.45) | -1.45±0.91 | 1.10E-01 | 4.15±3.05 | 1.74E-01 | 160(28.67) | -2.06±1.22 | 9.25E-02 | 7.47±4.88 | 1.27E-01 |
| Heart failure | 87(8.62) | -10.47±1.40 | 1.73E-13 | 19.24±4.82 | 7.22E-05 | 253(45.34) | -5.08±1.07 | 2.70E-06 | 14.45±4.21 | 7.00E-04 |
| Hypertension | 605(59.90) | 2.29±0.83 | 6.01E-03 | 12.76±2.75 | 4.13E-06 | 334(59.86) | 0.45±1.12 | 6.91E-01 | 10.36±4.32 | 1.72E-02 |
| Hyperlipidemia | 112(11.09) | 2.48±1.33 | 6.22E-02 | -1.78±4.52 | 6.94E-01 | 75(13.44) | 3.44±1.60 | 3.20E-02 | -8.50±6.09 | 1.64E-01 |
| **Biochemical measurements** |  |  |  |  |  |  |  |  |  |  |
| ALT,U/L | 27.62 ±15.08 | -0.11±0.03 | 6.58E-04 | 0.18±0.11 | 9.65E-02 | 27.79±24.70 | -0.02±0.02 | 3.79E-01 | -0.04±0.12 | 7.26E-01 |
| AST,U/L | 26.83 ±12.13 | -0.17±0.04 | 1.29E-05 | 0.42±0.13 | 1.58E-03 | 32.30±55.86 | -0.03±0.01 | 2.25E-02 | 0.16±0.11 | 1.53E-01 |
| eGFR,ml/min/1.73 m² | 95.02 ±74.57 | 0.01±0.01 | 1.18E-01 | -0.01±0.02 | 5.67E-01 | 91.50±111.62 | 0.01±0.00 | 1.53E-01 | -0.02±0.02 | 1.96E-01 |
| CK,U/L | 112.76 ±112.54 | 0.00±0.00 | 2.94E-01 | 0.02±0.01 | 7.38E-02 | 164.06±455.73 | 0.00±0.00 | 8.96E-01 | -0.01±0.02 | 5.54E-01 |
| CKMB,U/L | 7.54 ±5.92 | -0.09±0.07 | 2.27E-01 | 0.45±0.34 | 1.91E-01 | 19.50±53.20 | 0.01±0.01 | 3.06E-01 | 0.19±0.21 | 3.61E-01 |
| CHOL,mmol/L | 4.28 ±1.12 | 0.41±0.37 | 2.76E-01 | -0.92±1.23 | 4.55E-01 | 4.29±1.78 | 0.39±0.29 | 1.71E-01 | -1.10±1.01 | 2.77E-01 |
| LDLC,mmol/L | 2.58 ±0.90 | -0.04±0.47 | 9.38E-01 | -1.61±1.53 | 2.94E-01 | 2.71±1.01 | 1.21±0.57 | 3.39E-02 | -2.05±2.36 | 3.86E-01 |
| HDLC,mmol/L | 0.97 ±0.26 | 3.56±1.69 | 3.53E-02 | -6.41±5.56 | 2.49E-01 | 0.99±0.25 | 4.45±2.25 | 4.86E-02 | -15.18±9.49 | 1.11E-01 |
| GLUC,mmol/L | 6.70 ±2.69 | -0.42±0.15 | 5.15E-03 | -0.04±0.51 | 9.33E-01 | 6.03±2.19 | -0.66±0.27 | 1.32E-02 | 2.10±1.04 | 4.56E-02 |
| Lpa,mg/L | 304.64 ±321.15 | 0.00±0.00 | 3.72E-01 | 0.00±0.00 | 4.91E-01 | 286.06±325.57 | 0.00±0.00 | 3.27E-01 | 0.01±0.01 | 1.86E-01 |
| APOA,g/L | 1.05 ±0.28 | 5.75±1.68 | 6.52E-04 | -9.26±5.81 | 1.11E-01 | 1.15±0.25 | 1.66±2.51 | 5.08E-01 | -12.78±8.78 | 1.47E-01 |
| BNP,pg/mL | 5.45 ±1.63 | -4.03±0.26 | 1.37E-45 | 6.81±0.96 | 4.96E-12 | 2.26±0.75 | -8.75±0.60 | 3.39E-39 | 28.13±2.67 | 6.46E-22 |
| TRIG,mmol/L | 1.61 ±1.15 | 0.67±0.35 | 5.67E-02 | 0.76±1.16 | 5.13E-01 | 1.85±1.86 | 0.43±0.27 | 1.22E-01 | 0.31±0.96 | 7.51E-01 |
| **Medication** |  |  |  |  |  |  |  |  |  |  |
| β-blockers | 895(88.70) | -3.49±1.33 | 8.70E-03 | 1.00±4.38 | 8.19E-01 | 470(84.23) | -2.93±1.48 | 4.85E-02 | 11.80±5.81 | 4.32E-02 |
| ACEIs | 622(61.65) | -0.99±0.85 | 2.48E-01 | 2.12±2.89 | 4.63E-01 | 283(50.72) | -1.80±1.08 | 9.54E-02 | 11.36±4.19 | 7.08E-03 |
| CCBs | 278(27.55) | 2.93±0.90 | 1.13E-03 | 11.48±2.97 | 1.20E-04 | 161(28.85) | 3.44±1.17 | 3.50E-03 | 7.38±4.48 | 1.01E-01 |
| PPIs | 490(48.56) | 0.21±0.82 | 7.98E-01 | 3.47±2.73 | 2.04E-01 | 376(67.38) | 1.14±1.14 | 3.19E-01 | 5.51±4.46 | 2.18E-01 |
| **SYNTAX score** | 16.35 ±10.67 | -0.24±0.04 | 7.23E-11 | 0.32±0.13 | 1.25E-02 | 16.53±13.11 | -0.26±0.04 | 3.08E-10 | 0.72±0.16 | 8.71E-06 |
| Estimates were calculated by applying a linear regression model. Variables with *P* < 0.05 were included into multivariable analysis as covariates. LVEF = left ventricular ejection fraction; LVMI = left ventricular mass index; SE = standard error; other abbreviations as in Table 1. | | | | | | | | | | |

**Table S3. Relationship between lipid species and death in univariate and adjusted Cox analyses of the internal training cohort**

| **Lipid Species** | **Univariate Cox Regression** | | | **Adjusted Cox Analysis** | | |
| --- | --- | --- | --- | --- | --- | --- |
|  | **HR (95%CI)** | **P** | **FDR** | **HR (95%CI)** | **P** | **FDR** |
| LPC(16:0/0:0) | 0.24(0.12-0.47) | 3.36E-05 | 9.74E-04 | 0.35(0.18-0.70) | 2.81E-03 | 1.33E-02 |
| LPC(16:1/0:0) | 0.38(0.21-0.68) | 1.20E-03 | 1.48E-02 | 0.54(0.31-0.96) | 3.62E-02 | 6.53E-02 |
| LPC(18:2/0:0) | 0.22(0.12-0.41) | 1.00E-06 | 7.41E-05 | 0.25(0.13-0.46) | 9.71E-06 | 4.13E-04 |
| LPC(18:3/0:0) | 0.34(0.20-0.59) | 1.17E-04 | 2.60E-03 | 0.50(0.29-0.86) | 1.13E-02 | 3.22E-02 |
| LPC(20:3/0:0) | 0.28(0.16-0.51) | 2.28E-05 | 8.45E-04 | 0.40(0.22-0.71) | 1.64E-03 | 8.44E-03 |
| LPC(20:4/0:0) | 0.49(0.33-0.72) | 2.38E-04 | 3.97E-03 | 0.62(0.43-0.90) | 1.23E-02 | 3.44E-02 |
| LPC(22:6/0:0) | 0.24(0.13-0.46) | 1.63E-05 | 6.80E-04 | 0.28(0.14-0.56) | 2.52E-04 | 2.42E-03 |
| LPE(0:0/24:0) | 1.62(1.25-2.10) | 2.64E-04 | 4.19E-03 | 1.61(1.21-2.15) | 1.16E-03 | 6.83E-03 |
| LPE(0:0/24:1) | 1.78(1.28-2.47) | 5.46E-04 | 7.91E-03 | 1.69(1.17-2.45) | 5.46E-03 | 1.90E-02 |
| LPE(0:0/18:2) | 0.48(0.29-0.81) | 5.25E-03 | 4.32E-02 | 0.48(0.28-0.81) | 6.32E-03 | 2.12E-02 |
| LPE(0:0/18:3) | 0.49(0.30-0.81) | 4.96E-03 | 4.24E-02 | 0.60(0.37-0.96) | 3.26E-02 | 6.14E-02 |
| LPE(0:0/22:6) | 0.25(0.13-0.46) | 9.13E-06 | 5.07E-04 | 0.24(0.13-0.46) | 1.73E-05 | 5.16E-04 |
| LPE(0:0/24:6) | 0.21(0.10-0.47) | 1.13E-04 | 2.60E-03 | 0.13(0.05-0.34) | 2.57E-05 | 6.38E-04 |
| LPS(16:0/0:0) | 0.27(0.13-0.55) | 3.17E-04 | 4.81E-03 | 0.37(0.19-0.74) | 4.81E-03 | 1.81E-02 |
| PC(20:0/20:0) | 1.38(1.13-1.67) | 1.15E-03 | 1.45E-02 | 1.53(1.22-1.91) | 2.21E-04 | 2.27E-03 |
| PC(12:0/14:1) | 1.48(1.16-1.88) | 1.35E-03 | 1.61E-02 | 1.43(1.10-1.86) | 6.80E-03 | 2.25E-02 |
| PC(20:1/20:1) | 1.57(1.27-1.94) | 3.07E-05 | 9.70E-04 | 1.68(1.29-2.19) | 1.26E-04 | 1.69E-03 |
| PC(22:1/22:1) | 1.35(1.12-1.64) | 2.09E-03 | 2.28E-02 | 1.32(1.08-1.60) | 5.83E-03 | 2.00E-02 |
| PC(16:1/22:2) | 0.13(0.04-0.39) | 2.70E-04 | 4.19E-03 | 0.13(0.04-0.38) | 2.41E-04 | 2.40E-03 |
| PC(22:1/22:2) | 0.40(0.23-0.70) | 1.24E-03 | 1.50E-02 | 0.34(0.19-0.62) | 4.30E-04 | 3.77E-03 |
| PC(14:1/18:4) | 0.40(0.22-0.71) | 1.88E-03 | 2.13E-02 | 0.37(0.20-0.68) | 1.44E-03 | 7.79E-03 |
| PC(20:2/20:3) | 0.98(0.96-0.99) | 3.08E-03 | 3.02E-02 | 0.98(0.96-0.99) | 7.64E-03 | 2.40E-02 |
| PC(18:0/22:6) | 0.39(0.23-0.67) | 5.96E-04 | 8.46E-03 | 0.47(0.28-0.82) | 7.19E-03 | 2.35E-02 |
| PC(20:1/20:5) | 0.41(0.24-0.70) | 1.05E-03 | 1.37E-02 | 0.50(0.29-0.87) | 1.37E-02 | 3.72E-02 |
| PC(18:3/20:4) | 0.97(0.94-0.99) | 2.47E-03 | 2.62E-02 | 0.97(0.95-0.99) | 1.08E-02 | 3.10E-02 |
| PC(20:2/22:6) | 0.97(0.95-0.99) | 2.92E-03 | 3.00E-02 | 0.96(0.95-0.98) | 4.62E-04 | 3.93E-03 |
| PE(32:0) | 1.37(1.21-1.55) | 5.61E-07 | 5.35E-05 | 1.40(1.23-1.59) | 3.39E-07 | 2.02E-05 |
| PE(30:1) | 1.47(1.15-1.86) | 1.79E-03 | 2.06E-02 | 1.47(1.09-2.00) | 1.30E-02 | 3.58E-02 |
| PE(34:1) | 1.85(1.40-2.43) | 1.31E-05 | 6.24E-04 | 1.90(1.42-2.54) | 1.51E-05 | 5.16E-04 |
| PE(42:1) | 1.34(1.13-1.60) | 7.30E-04 | 9.73E-03 | 1.43(1.19-1.73) | 1.44E-04 | 1.72E-03 |
| PE(30:2) | 2.86(2.09-3.92) | 5.31E-11 | 1.18E-08 | 2.57(1.80-3.67) | 1.82E-07 | 1.36E-05 |
| PE(32:2) | 1.74(1.39-2.16) | 9.08E-07 | 7.41E-05 | 1.60(1.26-2.04) | 1.30E-04 | 1.69E-03 |
| PE(34:3) | 1.47(1.14-1.90) | 2.91E-03 | 3.00E-02 | 1.42(1.06-1.91) | 1.83E-02 | 4.43E-02 |
| CerP(d18:1/18:0) | 1.43(1.20-1.70) | 7.43E-05 | 1.87E-03 | 1.35(1.13-1.62) | 9.57E-04 | 6.31E-03 |
| CerP(d18:1/18:1) | 1.53(1.22-1.93) | 2.70E-04 | 4.19E-03 | 1.46(1.12-1.90) | 4.67E-03 | 1.78E-02 |
| Cer(d18:1/16:1) | 1.82(1.42-2.33) | 1.87E-06 | 1.25E-04 | 1.84(1.41-2.39) | 6.87E-06 | 3.41E-04 |
| Cer(d18:1/20:1) | 1.61(1.43-1.81) | 3.43E-15 | 2.29E-12 | 1.54(1.35-1.76) | 3.32E-10 | 7.61E-08 |
| Cer(d18:1/24:1) | 2.13(1.73-2.60) | 3.56E-13 | 1.19E-10 | 1.93(1.57-2.38) | 5.11E-10 | 7.61E-08 |
| Cer(d18:0/24:0) | 1.15(1.05-1.26) | 3.70E-03 | 3.52E-02 | 1.20(1.07-1.34) | 1.38E-03 | 7.60E-03 |
| Cer(m18:0/22:2) | 1.86(1.35-2.56) | 1.26E-04 | 2.60E-03 | 1.77(1.29-2.45) | 4.91E-04 | 4.07E-03 |
| Cer(t18:0/24:1) | 1.47(1.21-1.78) | 1.23E-04 | 2.60E-03 | 1.47(1.16-1.85) | 1.16E-03 | 6.83E-03 |
| HexCer(d18:1/22:0) | 1.72(1.34-2.19) | 1.41E-05 | 6.27E-04 | 1.64(1.25-2.15) | 3.72E-04 | 3.36E-03 |
| HexCer(d18:1/24:0) | 1.47(1.24-1.75) | 1.11E-05 | 5.70E-04 | 1.47(1.23-1.75) | 1.68E-05 | 5.16E-04 |
| HexCer(d18:1/26:0) | 1.66(1.31-2.10) | 2.04E-05 | 8.00E-04 | 1.66(1.28-2.15) | 1.15E-04 | 1.69E-03 |
| HexCer(d18:1/16:1) | 1.71(1.30-2.26) | 1.51E-04 | 2.80E-03 | 1.60(1.16-2.19) | 3.71E-03 | 1.51E-02 |
| HexCer(d18:1/18:1) | 1.47(1.22-1.77) | 3.96E-05 | 1.06E-03 | 1.27(1.04-1.54) | 1.95E-02 | 4.60E-02 |
| HexCer(d18:1/20:1) | 1.55(1.35-1.79) | 6.00E-10 | 8.00E-08 | 1.39(1.19-1.64) | 5.11E-05 | 9.52E-04 |
| HexCer(d18:1/24:1) | 1.48(1.27-1.72) | 4.14E-07 | 4.60E-05 | 1.33(1.12-1.57) | 8.59E-04 | 6.24E-03 |
| HexCer(d18:1/26:1) | 1.50(1.18-1.91) | 1.09E-03 | 1.39E-02 | 1.39(1.07-1.82) | 1.51E-02 | 3.92E-02 |
| PC(O-32:0) | 1.78(1.32-2.39) | 1.28E-04 | 2.60E-03 | 1.68(1.24-2.29) | 9.48E-04 | 6.31E-03 |
| PC(O-34:0) | 1.46(1.12-1.91) | 5.02E-03 | 4.24E-02 | 1.35(1.02-1.79) | 3.85E-02 | 6.62E-02 |
| PC(O-38:2) | 0.36(0.21-0.65) | 5.41E-04 | 7.91E-03 | 0.32(0.18-0.57) | 1.20E-04 | 1.69E-03 |
| PC(O-42:3) | 1.92(1.46-2.51) | 2.17E-06 | 1.32E-04 | 1.63(1.22-2.17) | 9.36E-04 | 6.31E-03 |
| PC(O-44:3) | 1.00(1.00-1.00) | 3.77E-03 | 3.55E-02 | 1.00(1.00-1.00) | 9.01E-04 | 6.31E-03 |
| PC(O-36:4) | 0.28(0.15-0.53) | 7.56E-05 | 1.87E-03 | 0.31(0.16-0.63) | 9.74E-04 | 6.31E-03 |
| PC(O-38:5) | 0.39(0.21-0.71) | 2.28E-03 | 2.45E-02 | 0.37(0.19-0.73) | 3.81E-03 | 1.53E-02 |
| PC(O-38:6) | 0.43(0.24-0.78) | 5.25E-03 | 4.32E-02 | 0.32(0.17-0.62) | 7.03E-04 | 5.24E-03 |
| PC(O-36:7) | 1.00(0.99-1.00) | 1.46E-04 | 2.80E-03 | 1.00(0.99-1.00) | 1.01E-03 | 6.40E-03 |
| PE(P-40:0) | 1.40(1.26-1.55) | 2.20E-10 | 3.67E-08 | 1.39(1.24-1.56) | 1.66E-08 | 1.65E-06 |
| PE(P-44:3) | 1.44(1.15-1.81) | 1.59E-03 | 1.86E-02 | 1.51(1.21-1.88) | 2.02E-04 | 2.15E-03 |
| PE(P-44:6) | 1.35(1.09-1.67) | 5.57E-03 | 4.47E-02 | 1.36(1.08-1.72) | 9.48E-03 | 2.85E-02 |
| PE(P-40:7) | 0.45(0.26-0.76) | 3.18E-03 | 3.07E-02 | 0.33(0.18-0.60) | 3.26E-04 | 3.04E-03 |
| LPC(O-18:0) | 0.35(0.17-0.70) | 2.98E-03 | 3.01E-02 | 0.50(0.25-0.97) | 4.08E-02 | 6.87E-02 |
| LPC(O-16:1) | 0.31(0.17-0.57) | 1.51E-04 | 2.80E-03 | 0.45(0.25-0.82) | 9.15E-03 | 2.81E-02 |
| MG(14:0) | 0.24(0.13-0.47) | 2.62E-05 | 9.20E-04 | 0.26(0.13-0.49) | 5.03E-05 | 9.52E-04 |
| MG(16:0) | 0.20(0.09-0.43) | 3.20E-05 | 9.70E-04 | 0.18(0.08-0.41) | 3.22E-05 | 7.38E-04 |
| MG(18:1) | 0.00(0.00-0.01) | 3.73E-05 | 1.04E-03 | 0.00(0.00-0.00) | 3.83E-05 | 8.15E-04 |
| DG(14:0/22:0/0:0) | 0.50(0.31-0.81) | 4.79E-03 | 4.18E-02 | 0.48(0.29-0.80) | 4.98E-03 | 1.85E-02 |
| DG(14:0/16:1/0:0) | 0.08(0.02-0.30) | 1.63E-04 | 2.93E-03 | 0.07(0.02-0.28) | 1.90E-04 | 2.10E-03 |
| DG(16:1/18:0/0:0) | 0.30(0.15-0.60) | 6.99E-04 | 9.51E-03 | 0.30(0.14-0.62) | 1.18E-03 | 6.83E-03 |
| DG(14:1/20:0/0:0) | 0.22(0.11-0.45) | 3.19E-05 | 9.70E-04 | 0.23(0.11-0.47) | 6.49E-05 | 1.14E-03 |
| DG(16:1/20:0/0:0) | 0.16(0.06-0.40) | 8.64E-05 | 2.06E-03 | 0.14(0.05-0.38) | 1.02E-04 | 1.59E-03 |
| TG(16:0/18:3/22:0) | 1.00(1.00-1.01) | 4.08E-03 | 3.78E-02 | 1.01(1.00-1.01) | 1.74E-04 | 1.99E-03 |
| TG(14:1/14:1/20:2) | 1.34(1.11-1.61) | 1.98E-03 | 2.20E-02 | 1.44(1.19-1.73) | 1.43E-04 | 1.72E-03 |
| TG(14:1/14:1/22:2) | 1.50(1.13-1.99) | 4.83E-03 | 4.18E-02 | 1.55(1.15-2.10) | 4.32E-03 | 1.69E-02 |
| TG(14:0/18:0/22:4) | 1.33(1.13-1.56) | 6.17E-04 | 8.57E-03 | 1.39(1.20-1.62) | 2.19E-05 | 5.93E-04 |
| TG(14:1/14:1/22:3) | 1.49(1.21-1.83) | 1.95E-04 | 3.33E-03 | 1.57(1.26-1.96) | 7.35E-05 | 1.22E-03 |
| PS(32:3) | 0.42(0.23-0.76) | 4.37E-03 | 3.94E-02 | 0.43(0.23-0.78) | 6.10E-03 | 2.06E-02 |
| Variables with *FDR* < 0.05 in univariate Cox regression were included into adjusted Cox analysis, and only variables with adjusted *P* < 0.05 were included into the lasso Cox model. In adjusted analysis, eGFR, age, glucose, AST, diabetes and heart failure status, CCB and PPIs treatment, and SYNTAX score were used as covariates. Finally, 78 lipid species were associated with all-cause mortality (*FDR* < 0.05) in univariate Cox regression and adjusted Cox analysis (*P* < 0.05) at the same time. *FDR* = false discovery rate. | | | | | | |

**Table S4. Relationship between lipid species and MACE in univariate and adjusted Cox analyses of the internal training cohort**

| **Lipid Species** | **Univariate Cox Regression** | | | **Adjusted Cox Regression** | | |
| --- | --- | --- | --- | --- | --- | --- |
|  | **HR (95%CI)** | **P** | **FDR** | **HR (95%CI)** | **P** | **FDR** |
| LPC(20:0/0:0) | 0.59(0.44-0.80) | 6.13E-04 | 8.90E-02 | 0.64(0.47-0.86) | 2.83E-03 | 6.56E-02 |
| LPC(18:2/0:0) | 0.57(0.43-0.76) | 1.32E-04 | 4.40E-02 | 0.64(0.48-0.85) | 2.27E-03 | 6.56E-02 |
| Cer(d18:1/20:1) | 1.26(1.13-1.40) | 3.63E-05 | 2.42E-02 | 1.15(1.03-1.30) | 1.57E-02 | 8.68E-02 |
| HexCer(d18:1/18:1) | 1.26(1.10-1.43) | 5.98E-04 | 8.90E-02 | 1.17(1.02-1.34) | 2.53E-02 | 9.20E-02 |
| HexCer(d18:1/20:1) | 1.24(1.09-1.40) | 6.67E-04 | 8.90E-02 | 1.14(1.01-1.30) | 4.17E-02 | 9.82E-02 |
| Variables with *FDR* < 0.1 in univariate Cox regression were used for adjusted analysis, and only variables with adjusted *P* < 0.05 were included into the multivariable Cox model. In adjusted analysis, AST, diabetes, arrhythmia, and heart failure status, CCB and PPIs treatment, and SYNTAX score were used as covariates. Finally, 5 lipid species were associated with all-cause mortality (*FDR* < 0.1) in univariate Cox regression and adjusted analysis (*P* < 0.05) at the same time. FDR = false discovery rate. | | | | | | |

| **Table S5. Relationship between lipid species and death in univariate and adjusted Cox analyses of external validation cohort** | | | | | | |
| --- | --- | --- | --- | --- | --- | --- |
| **Lipid Species** | **Univariate Cox Regression** | | | **Adjusted Cox Analysis** | | |
|  | **HR (95%CI)** | **P** | **FDR** | **HR (95%CI)** | **P** | **FDR** |
| LPC(16:0/0:0) | 0.2(0.08-0.52) | 9.05E-04 | 1.85E-02 | 0.34(0.13-0.87) | 2.42E-02 | 1.04E-01 |
| LPS(16:0/0:0) | 0.19(0.07-0.52) | 1.09E-03 | 1.85E-02 | 0.30(0.11-0.82) | 1.90E-02 | 1.04E-01 |
| LPC(20:3/0:0) | 0.26(0.11-0.64) | 3.45E-03 | 3.91E-02 | 0.41(0.17-1.00) | 4.97E-02 | 1.04E-01 |
| LPE(0:0/22:6) | 0.29(0.12-0.70) | 6.08E-03 | 4.64E-02 | 0.46(0.18-1.12) | 8.66E-02 | 1.04E-01 |
| LPC(22:6/0:0) | 0.28(0.11-0.71) | 7.68E-03 | 4.64E-02 | 0.39(0.15-1.04) | 5.98E-02 | 1.04E-01 |
| CerP(d18:1/18:1) | 1.56(1.12-2.16) | 8.18E-03 | 4.64E-02 | 1.38(0.97-1.97) | 7.68E-02 | 1.04E-01 |
| PC(16:1/22:2) | 0.34(0.15-0.81) | 1.41E-02 | 6.85E-02 | 0.46(0.19-1.12) | 8.66E-02 | 1.04E-01 |
| LPC(20:4/0:0) | 0.40(0.18-0.90) | 2.63E-02 | 1.12E-01 |  |  |  |
| LPC(18:2/0:0) | 0.43(0.20-0.93) | 3.14E-02 | 1.19E-01 |  |  |  |
| PC(18:0/22:6) | 0.40(0.17-0.96) | 3.93E-02 | 1.24E-01 | 0.45(0.18-1.15) | 9.54E-02 | 1.04E-01 |
| LPC(16:1/0:0) | 0.40(0.17-0.97) | 4.18E-02 | 1.24E-01 |  |  |  |
| LPE(0:0/24:1) | 1.58(1.01-2.46) | 4.38E-02 | 1.24E-01 | 1.60(1.05-2.43) | 2.79E-02 | 1.04E-01 |
| LPC(18:3/0:0) | 0.44(0.19-1.01) | 5.41E-02 | 1.41E-01 | 0.46(0.20-1.08) | 7.41E-02 | 1.04E-01 |
| Cer(d18:1/20:1) | 1.48(0.94-2.34) | 8.83E-02 | 2.14E-01 |  |  |  |
| PC(20:1/20:5) | 0.50(0.23-1.12) | 9.42E-02 | 2.14E-01 |  |  |  |
| Cer(d18:1/16:1) | 1.69(0.89-3.21) | 1.08E-01 | 2.30E-01 | 1.95(1.00-3.79) | 4.98E-02 | 1.04E-01 |
| Cer(d18:1/24:1) | 1.44(0.91-2.30) | 1.23E-01 | 2.46E-01 |  |  |  |
| LPE(0:0/18:3) | 0.58(0.28-1.22) | 1.52E-01 | 2.87E-01 |  |  |  |
| DG(14:1/20:0/0:0) | 1.38(0.76-2.52) | 2.86E-01 | 4.81E-01 |  |  |  |
| DG(16:1/20:0/0:0) | 1.33(0.79-2.23) | 2.89E-01 | 4.81E-01 |  |  |  |
| TG(14:1/14:1/22:3) | 0.64(0.27-1.49) | 2.97E-01 | 4.81E-01 |  |  |  |
| LPE(0:0/18:2) | 0.68(0.32-1.46) | 3.24E-01 | 5.01E-01 |  |  |  |
| DG(16:1/18:0/0:0) | 1.34(0.70-2.57) | 3.80E-01 | 5.57E-01 |  |  |  |
| TG(14:1/14:1/22:2) | 0.70(0.31-1.59) | 3.93E-01 | 5.57E-01 |  |  |  |
| Cer(d18:0/24:0) | 0.83(0.51-1.37) | 4.70E-01 | 6.21E-01 |  |  |  |
| TG(14:0/18:0/22:4) | 0.72(0.29-1.80) | 4.80E-01 | 6.21E-01 |  |  |  |
| LPE(0:0/24:6) | 0.89(0.62-1.26) | 5.07E-01 | 6.21E-01 |  |  |  |
| PC(20:2/22:6) | 1.26(0.63-2.51) | 5.16E-01 | 6.21E-01 |  |  |  |
| PC(22:1/22:2) | 1.18(0.71-1.96) | 5.30E-01 | 6.21E-01 |  |  |  |
| TG(14:1/14:1/20:2) | 0.87(0.50-1.52) | 6.18E-01 | 7.00E-01 |  |  |  |
| HexCer(d18:1/22:0) | 1.06(0.81-1.38) | 6.93E-01 | 7.60E-01 |  |  |  |
| LPE(0:0/24:0) | 1.03(0.57-1.84) | 9.32E-01 | 9.60E-01 |  |  |  |
| DG(14:0/16:1/0:0) | 0.98(0.59-1.65) | 9.46E-01 | 9.60E-01 | 0.39(0.09-1.81) | 2.32E-01 | 2.32E-01 |
| CerP(d18:1/18:0) | 1.01(0.61-1.69) | 9.60E-01 | 9.60E-01 |  |  |  |
| HRs (95% CI) were calculated by applying a Cox regression model. In adjusted analysis, age, AST, creatine kinase and eGFR levels were used as covariates. | | | | | | |

**Table S6. Disturbed metabolic pathways in CAD patients with higher risks from death**

| **Pathway Name** | **Total** | **Hits** | **P** | **Impact** | **Hits metabolites** |
| --- | --- | --- | --- | --- | --- |
| Glycerophospholipid metabolism | 39 | 3 | 7.6E-05 | 0.23123 | LysoPC (18:2(9Z,12Z)) PC(16:1(9Z)/22:2(13Z,16Z))  PE(O-18:1(1Z)/20:4(5Z,8Z,11Z,14Z)) |
| Glycosylphosphatidylinositol (GPI)-anchor biosynthesis | 14 | 1 | 0.03443 | 0.0439 | PE(O-18:1(1Z)/20:4(5Z,8Z,11Z,14Z)) |
| Linoleic acid metabolism | 15 | 1 | 0.03685 | 0 | PC(16:0/16:0) |
| Ether lipid metabolism | 23 | 1 | 0.05604 | 0.04204 | LysoPC(O-18:0) |
| Sphingolipid metabolism | 25 | 1 | 0.06078 | 0.29423 | Cer(d18:1/24:1(15Z)) |
| alpha-Linolenic acid metabolism | 29 | 1 | 0.07022 | 0 | PC(16:0/16:0) |
| Arachidonic acid metabolism | 62 | 1 | 0.14507 | 0 | PC(16:0/16:0) |

**Table S7. Multivariable Cox proportional hazard model for MACE**

| **Terms** | **HR (95% CI)** | **P value** |
| --- | --- | --- |
| arrhythmia | 1.48(0.93-2.36) | 9.67E-02 |
| DM | 1.35(0.98-1.86) | 6.87E-02 |
| HF | 1.88(1.24-2.85) | 2.72E-03 |
| SYNTAX | 1.02(1.00-1.03) | 9.46E-03 |
| CCB | 1.39(1.00-1.91) | 4.79E-02 |
| PPI | 1.65(1.20-2.27) | 2.16E-03 |
| Cer(d18:1/20:1) | 1.14(1.02-1.28) | 2.56E-02 |
| LPC(20:0/0:0) | 0.65(0.48-0.88) | 4.60E-03 |
| HRs (95% CI) were calculated by applying a Cox regression model. Variables with adjusted *P* < 0.05 were included into the multivariable Cox regression analysis. When fitting the multivariable Cox proportional hazards model, a backward stepwise process based on AIC (akaike information criterion) was used to refine variables, and model with the smallest AIC value was consider the best. Abbreviations as in Table 1. | | |

**Table S8. Lipid species associated with ACS (vs. stable CAD) in the internal training cohort**

| **Lipid Species** | **Univariate Logistic Regression** | | | **Adjusted Logistic Regression** | | |
| --- | --- | --- | --- | --- | --- | --- |
|  | **OR (95%CI)** | **P** | **FDR** | **OR (95%CI)** | **P** | **FDR** |
| PC(18:2/20:4) | 0.57(0.44-0.72) | 3.66E-06 | 1.22E-03 | 0.62(0.48-0.80) | 2.56E-04 | 5.15E-02 |
| LPC(18:2/0:0) | 0.57(0.46-0.71) | 2.18E-07 | 1.45E-04 | 0.66(0.52-0.83) | 3.21E-04 | 3.23E-02 |
| PC(O-38:2) | 0.64(0.51-0.80) | 9.37E-05 | 1.04E-02 | 0.74(0.58-0.95) | 1.87E-02 | 6.97E-02 |
| LPC(20:0/0:0) | 0.66(0.54-0.79) | 1.72E-05 | 3.82E-03 | 0.71(0.58-0.86) | 6.61E-04 | 4.43E-02 |
| LPC(18:1/0:0) | 0.67(0.54-0.83) | 3.11E-04 | 1.88E-02 | 0.71(0.56-0.89) | 3.21E-03 | 6.45E-02 |
| PE(P-42:5) | 0.67(0.54-0.83) | 2.80E-04 | 1.87E-02 | 0.79(0.63-1.00) | 4.95E-02 | 1.21E-01 |
| LPC(16:0/0:0) | 0.68(0.54-0.85) | 8.75E-04 | 2.24E-02 | 0.70(0.55-0.88) | 3.06E-03 | 6.84E-02 |
| LPC(22:0/0:0) | 0.68(0.57-0.81) | 2.58E-05 | 4.30E-03 | 0.73(0.60-0.88) | 1.27E-03 | 5.12E-02 |
| PC(18:2/18:2) | 0.69(0.56-0.85) | 5.07E-04 | 2.11E-02 | 0.76(0.61-0.96) | 2.11E-02 | 7.20E-02 |
| LPE(P-20:0) | 0.69(0.56-0.86) | 8.53E-04 | 2.37E-02 | 0.74(0.59-0.92) | 8.06E-03 | 7.36E-02 |
| PC(20:0/20:4) | 0.70(0.56-0.87) | 1.40E-03 | 3.00E-02 | 0.78(0.61-0.98) | 3.10E-02 | 9.30E-02 |
| LPE(0:0/20:1) | 0.70(0.57-0.85) | 5.15E-04 | 2.02E-02 | 0.73(0.59-0.90) | 3.39E-03 | 5.69E-02 |
| LPC(O-16:1) | 0.70(0.57-0.86) | 6.81E-04 | 2.27E-02 | 0.77(0.62-0.95) | 1.75E-02 | 6.75E-02 |
| LPC(18:3/0:0) | 0.70(0.58-0.83) | 9.71E-05 | 9.25E-03 | 0.75(0.62-0.90) | 2.81E-03 | 8.06E-02 |
| LPC(20:3/0:0) | 0.70(0.58-0.84) | 1.91E-04 | 1.42E-02 | 0.73(0.60-0.88) | 1.42E-03 | 4.76E-02 |
| LPE(0:0/18:2) | 0.70(0.58-0.85) | 3.53E-04 | 1.96E-02 | 0.76(0.62-0.92) | 6.75E-03 | 7.14E-02 |
| LPC(20:2/0:0) | 0.71(0.58-0.85) | 4.25E-04 | 2.03E-02 | 0.75(0.61-0.91) | 4.08E-03 | 5.85E-02 |
| LPE(0:0/20:0) | 0.72(0.59-0.86) | 6.81E-04 | 2.39E-02 | 0.75(0.61-0.90) | 4.62E-03 | 6.19E-02 |
| LPC(16:1/0:0) | 0.73(0.60-0.88) | 1.38E-03 | 3.07E-02 | 0.73(0.60-0.90) | 3.55E-03 | 5.48E-02 |
| LPC(O-20:0) | 0.73(0.61-0.87) | 4.88E-04 | 2.17E-02 | 0.79(0.66-0.95) | 1.41E-02 | 6.75E-02 |
| LPE(0:0/24:6) | 0.73(0.61-0.87) | 7.17E-04 | 2.28E-02 | 0.79(0.65-0.95) | 1.26E-02 | 6.83E-02 |
| LPC(22:6/0:0) | 0.73(0.61-0.88) | 7.75E-04 | 2.35E-02 | 0.78(0.65-0.94) | 1.01E-02 | 6.35E-02 |
| LPC(22:2/0:0) | 0.74(0.60-0.90) | 2.41E-03 | 3.65E-02 | 0.76(0.62-0.94) | 1.00E-02 | 6.50E-02 |
| LPC(O-18:0) | 0.74(0.61-0.88) | 8.62E-04 | 2.30E-02 | 0.78(0.65-0.94) | 9.70E-03 | 6.72E-02 |
| PE(42:4) | 0.76(0.63-0.89) | 1.16E-03 | 2.77E-02 | 0.78(0.65-0.92) | 5.62E-03 | 6.65E-02 |
| LPC(O-18:1) | 0.76(0.63-0.91) | 2.89E-03 | 4.01E-02 | 0.79(0.65-0.96) | 1.75E-02 | 6.64E-02 |
| PE(40:2) | 0.77(0.66-0.89) | 7.93E-04 | 2.30E-02 | 0.78(0.66-0.91) | 2.84E-03 | 7.15E-02 |
| TG(16:1/16:1/20:3) | 0.77(0.66-0.91) | 2.47E-03 | 3.66E-02 | 0.77(0.64-0.91) | 3.22E-03 | 5.88E-02 |
| LPC(20:4/0:0) | 0.78(0.68-0.88) | 6.55E-05 | 8.74E-03 | 0.80(0.70-0.91) | 6.82E-04 | 3.43E-02 |
| PE(44:4) | 0.79(0.68-0.91) | 2.03E-03 | 3.39E-02 | 0.82(0.70-0.95) | 9.50E-03 | 6.82E-02 |
| PE(P-38:3) | 0.84(0.75-0.93) | 1.89E-03 | 3.41E-02 | 0.88(0.78-0.99) | 3.38E-02 | 9.43E-02 |
| PE(42:2) | 0.84(0.75-0.94) | 2.41E-03 | 3.73E-02 | 0.85(0.76-0.95) | 7.25E-03 | 7.29E-02 |
| PG(34:0) | 0.99(0.98-0.99) | 6.03E-04 | 2.24E-02 | 0.99(0.98-1.00) | 1.47E-02 | 6.70E-02 |
| PC(18:3/20:4) | 0.99(0.98-1.00) | 1.22E-03 | 2.80E-02 | 0.99(0.98-1.00) | 1.69E-02 | 6.66E-02 |
| PE(44:3) | 0.99(0.98-1.00) | 2.63E-03 | 3.81E-02 | 0.99(0.99-1.00) | 1.06E-02 | 6.24E-02 |
| HexCer(d18:1/26:1) | 1.26(1.09-1.47) | 1.65E-03 | 3.25E-02 | 1.18(1.01-1.37) | 3.85E-02 | 1.05E-01 |
| PC(20:1/20:1) | 1.29(1.09-1.53) | 2.83E-03 | 4.02E-02 | 1.27(1.06-1.52) | 8.95E-03 | 7.20E-02 |
| HexCer(d18:1/24:0) | 1.29(1.14-1.48) | 1.25E-04 | 1.04E-02 | 1.19(1.04-1.37) | 1.21E-02 | 6.77E-02 |
| HexCer(d18:1/22:0) | 1.31(1.12-1.54) | 1.08E-03 | 2.66E-02 | 1.20(1.01-1.42) | 4.19E-02 | 1.09E-01 |
| Cer(d18:1/20:0) | 1.34(1.11-1.61) | 1.97E-03 | 3.36E-02 | 1.25(1.04-1.53) | 2.11E-02 | 7.30E-02 |

**Table S9. Disturbed metabolic pathways in patients with ACS vs. stable CAD**

| **Pathway Name** | **Total** | **Hits** | **P** | **Impact** | **Hit metabolites** |
| --- | --- | --- | --- | --- | --- |
| Glycerophospholipid metabolism | 36 | 3 | 1.12E-04 | 0.21631 | Phosphatidylethanolamine |
|  |  |  |  |  | Phosphatidylcholine |
|  |  |  |  |  | 1-Acyl-sn-glycero-3-phosphocholine |
| Linoleic acid metabolism | 5 | 1 | 1.60E-02 | 0 | Phosphatidylcholine |
| alpha-Linolenic acid metabolism | 13 | 1 | 4.13E-02 | 0 | Phosphatidylcholine |
| Glycosylphosphatidylinositol (GPI)-anchor biosynthesis | 14 | 1 | 4.44E-02 | 0.00399 | Phosphatidylethanolamine |
| Ether lipid metabolism | 20 | 1 | 6.30E-02 | 0.14458 | 1-Organyl-2-lyso-sn-glycero-3-phosphocholine |
| Sphingolipid metabolism | 21 | 1 | 6.60E-02 | 0.26978 | N-Acylsphingosine |
| Arachidonic acid metabolism | 36 | 1 | 1.11E-01 | 0 | Phosphatidylcholine |

**Table S10. Lipid species associated with ACS (vs. stable CAD) in the external validation cohort**

| **Lipid Species** | **Univariate Logistic Regression** | | | **Adjusted Logistic Regression** | | |
| --- | --- | --- | --- | --- | --- | --- |
|  | **OR (95%CI)** | **P** | **FDR** | **OR (95%CI)** | **P** | **FDR** |
| LPE(0:0/24:6) | 0.55(0.46-0.66) | 3.40E-10 | 1.05E-07 | 0.63(0.50-0.77) | 2.91E-05 | 6.21E-04 |
| LPC(18:3/0:0) | 1.90(1.51-2.44) | 1.44E-07 | 7.44E-06 | 1.94(1.44-2.67) | 2.48E-05 | 1.06E-03 |
| LPC(22:0/0:0) | 0.51(0.38-0.66) | 9.74E-07 | 3.76E-05 | 0.54(0.37-0.76) | 5.99E-04 | 2.74E-03 |
| LPC(22:6/0:0) | 0.70(0.54-0.89) | 5.10E-03 | 2.72E-02 |  |  |  |
| PC(18:2/18:2) | 0.74(0.56-0.96) | 2.62E-02 | 8.63E-02 | 0.64(0.43-0.93) | 2.02E-02 | 3.24E-02 |
| LPC(20:0/0:0) | 0.73(0.54-0.95) | 3.02E-02 | 9.44E-02 |  |  |  |
| LPC(22:2/0:0) | 0.73(0.53-0.95) | 3.33E-02 | 9.80E-02 |  |  |  |
| LPC(18:2/0:0) | 0.76(0.58-1.01) | 5.94E-02 | 1.40E-01 |  |  |  |
| LPC(20:2/0:0) | 0.79(0.61-1.01) | 7.25E-02 | 1.65E-01 |  |  |  |
| LPC(16:1/0:0) | 1.23(0.94-1.61) | 1.28E-01 | 2.28E-01 | 1.41(0.98-2.02) | 6.08E-02 | 7.70E-02 |
| HexCer(d18:1/22:0) | 1.13(0.97-1.38) | 1.64E-01 | 2.60E-01 |  |  |  |
| LPE(0:0/20:0) | 0.85(0.66-1.07) | 1.76E-01 | 2.73E-01 |  |  |  |
| LPC(20:4/0:0) | 0.88(0.68-1.12) | 3.10E-01 | 4.12E-01 |  |  |  |
| PC(18:2/20:4) | 0.89(0.68-1.15) | 3.81E-01 | 4.80E-01 |  |  |  |
| LPE(0:0/18:2) | 1.07(0.83-1.38) | 5.86E-01 | 6.73E-01 |  |  |  |
| LPC(20:3/0:0) | 0.94(0.73-1.20) | 6.13E-01 | 6.97E-01 |  |  |  |
| LPC(18:1/0:0) | 1.07(0.79-1.45) | 6.52E-01 | 7.22E-01 | 1.47(1.00-2.17) | 5.01E-02 | 6.54E-02 |
| LPC(16:0/0:0) | 1.06(0.79-1.43) | 6.75E-01 | 7.38E-01 | 1.54(1.04-2.29) | 2.71E-02 | 3.99E-02 |
| TG(16:1/16:1/20:3) | 1.02(0.82-1.26) | 8.50E-01 | 8.79E-01 |  |  |  |
| LPE(0:0/20:1) | 1.00(0.78-1.28) | 9.90E-01 | 9.93E-01 |  |  |  |

**Table S11. Association of the prognostic lipid species with LVEF and LVMI in the internal training cohort**

| **Lipid Species Associated with Clinical Outcomes** | **LVEF** | | | | **LVMI** | | | |
| --- | --- | --- | --- | --- | --- | --- | --- | --- |
|  | **Univariate Analysis** | | **Adjusted Analysis** | | **Univariate Analysis** | | **Adjusted Analysis** | |
|  | **Estimate ± SE** | **P value** | **Estimate ± SE** | **P value** | **Estimate ± SE** | **P value** | **Estimate ± SE** | **P value** |
| HexCer(d18:1/18:1) | -1.68±0.43 | 1.16E-04 | -2.10±0.46 | 4.78E-06 | 2.37±1.53 | 1.22E-01 | 2.62±1.58 | 9.77E-02 |
| PE(P-40:7) | 2.64±0.68 | 1.21E-04 | 1.75±0.68 | 1.06E-02 | -1.92±2.36 | 4.18E-01 |  |  |
| Cer(t18:0/24:1) | -1.83±0.48 | 1.28E-04 | -1.77±0.51 | 4.74E-04 | 1.04±1.74 | 5.52E-01 |  |  |
| HexCer(d18:1/22:0) | -1.87±0.49 | 1.62E-04 | -1.83±0.52 | 5.14E-04 | 7.29±1.84 | 8.18E-05 | 6.66±1.91 | 5.20E-04 |
| PC(20:1/20:1) | -1.98±0.53 | 2.02E-04 | -1.68±0.55 | 2.54E-03 | 2.09±1.91 | 2.73E-01 |  |  |
| CerP(d18:1/18:1) | -1.73±0.48 | 3.12E-04 | -2.12±0.48 | 1.39E-05 | 3.24±1.76 | 6.56E-02 | 3.07±1.78 | 8.47E-02 |
| PC(18:0/22:6) | 2.51±0.71 | 4.14E-04 | 1.47±0.71 | 3.88E-02 | -4.25±2.41 | 7.76E-02 |  |  |
| PC(20:1/20:5) | 2.48±0.70 | 4.25E-04 | 1.42±0.71 | 4.54E-02 | -3.38±2.38 | 1.57E-01 |  |  |
| PE(30:1) | -1.94±0.55 | 4.75E-04 | -1.65±0.60 | 6.22E-03 | 2.67±1.94 | 1.68E-01 |  |  |
| LPC(O-16:1) | 2.18±0.63 | 6.26E-04 | 1.71±0.64 | 7.46E-03 | -2.03±2.12 | 3.37E-01 |  |  |
| PC(O-32:0) | -2.18±0.68 | 1.41E-03 | -2.30±0.73 | 1.72E-03 | 3.98±2.50 | 1.12E-01 |  |  |
| HexCer(d18:1/26:1) | -1.46±0.46 | 1.45E-03 | -1.65±0.48 | 6.76E-04 | 4.43±1.62 | 6.51E-03 | 3.35±1.65 | 4.26E-02 |
| PC(O-38:2) | 2.24±0.71 | 1.63E-03 |  |  | -5.25±2.43 | 3.09E-02 |  |  |
| PC(14:1/18:4) | 1.63±0.53 | 2.17E-03 |  |  | -2.14±1.81 | 2.37E-01 |  |  |
| PE(34:1) | -1.74±0.57 | 2.51E-03 | -2.24±0.58 | 1.24E-04 | 6.48±2.01 | 1.32E-03 | 5.56±2.02 | 6.06E-03 |
| TG(14:1/14:1/20:2) | 1.19±0.40 | 2.98E-03 |  |  | 0.22±1.40 | 8.72E-01 |  |  |
| HexCer(d18:1/24:0) | -1.14±0.39 | 3.74E-03 | -1.50±0.42 | 4.21E-04 | 3.14±1.44 | 2.91E-02 |  |  |
| TG(14:1/14:1/22:3) | 1.25±0.43 | 3.99E-03 |  |  | 0.27±1.50 | 8.60E-01 |  |  |
| HexCer(d18:1/20:1) | -1.15±0.41 | 4.84E-03 | -1.37±0.42 | 1.22E-03 | 1.08±1.46 | 4.60E-01 |  |  |
| Cer(d18:1/20:1) | -1.07±0.38 | 5.30E-03 | -1.17±0.40 | 3.25E-03 | 1.39±1.35 | 3.05E-01 |  |  |
| Cer(d18:1/24:1) | -1.19±0.45 | 8.64E-03 | -1.14±0.47 | 1.46E-02 | 2.20±1.61 | 1.72E-01 |  |  |
| Cer(d18:1/16:1) | -1.47±0.57 | 9.26E-03 | -1.96±0.61 | 1.43E-03 | 1.72±1.97 | 3.83E-01 |  |  |
| LPC(18:3/0:0) | 1.42±0.56 | 1.17E-02 |  |  | -1.24±1.90 | 5.15E-01 |  |  |
| LPE(0:0/24:6) | 1.45±0.60 | 1.59E-02 | 1.05±0.60 | 8.02E-02 | -0.95±2.16 | 6.59E-01 |  |  |
| PC(O-34:0) | -1.19±0.50 | 1.85E-02 | -1.20±0.56 | 3.18E-02 | 2.91±1.79 | 1.05E-01 |  |  |
| LPC(18:2/0:0) | 1.51±0.66 | 2.31E-02 |  |  | -1.13±2.24 | 6.15E-01 |  |  |
| HexCer(d18:1/16:1) | -1.36±0.60 | 2.42E-02 | -1.74±0.65 | 7.37E-03 | 1.02±2.04 | 6.18E-01 |  |  |
| HexCer(d18:1/26:0) | -1.02±0.48 | 3.34E-02 | -1.53±0.52 | 3.02E-03 | 4.70±1.67 | 5.00E-03 | 4.05±1.78 | 2.29E-02 |
| PC(18:3/20:4) | 0.05±0.02 | 4.97E-02 |  |  | 0.04±0.08 | 6.10E-01 |  |  |
| Estimates were calculated by applying a linear regression model. The empty cells were the variables excluded in multivariate regression based on backward stepwise. The lipid species refer to the 79 metabolites associated with death and MACE (adjusted *P*< 0.05). In adjusted analysis, sex, arrhythmia, heart failure and hypertension statuses, glucose and HDLC levels CCBs and β-blockers treatment, ALT, AST and SYNTAX score were included as covariates to analyze the association of metabolites with LVEF; sex, arrhythmia, heart failure and hypertension statuses, CCBs treatment, AST and SYNTAX score were used as covariates to analyze the association of metabolites with LVMI. | | | | | | | | |

**Table S12. Association of the prognostic lipid species with LVEF and LVMI in the external validation cohort**

| **Lipid Species Associated with Clinical Outcomes** | **LVEF** | | | | **LVMI** | | | |
| --- | --- | --- | --- | --- | --- | --- | --- | --- |
|  | **Univariate Analysis** | | **Adjusted Analysis** | | **Univariate Analysis** | | **Adjusted Analysis** | |
|  | **Estimate ± SE** | **P value** | **Estimate ± SE** | **P value** | **Estimate ± SE** | **P value** | **Estimate ± SE** | **P value** |
| PC(20:1/20:5) | 3.23±0.85 | 1.68E-04 | 1.66±0.86 | 5.26E-02 | -4.17±3.51 | 2.35E-01 |  |  |
| PC(18:0/22:6) | 3.41±0.96 | 4.19E-04 |  |  | -5.71±3.92 | 1.46E-01 |  |  |
| CerP(d18:1/18:1) | -2.51±0.74 | 8.06E-04 | -2.06±0.75 | 6.15E-03 | 4.31±3.06 | 1.60E-01 |  |  |
| PC(16:1/22:2) | 2.68±0.93 | 4.13E-03 |  |  | -6.13±3.90 | 1.17E-01 |  |  |
| LPC(22:6/0:0) | 2.02±0.71 | 4.93E-03 |  |  | -5.90±2.98 | 4.89E-02 |  |  |
| LPE(0:0/24:6) | 1.00±0.38 | 8.59E-03 | 0.61±0.36 | 9.59E-02 | -1.33±1.42 | 3.48E-01 |  |  |
| LPE(0:0/22:6) | 2.19±0.86 | 1.13E-02 |  |  | -4.87±3.49 | 1.63E-01 |  |  |
| LPC(16:0/0:0) | 2.57±1.05 | 1.44E-02 |  |  | -10.89±4.48 | 1.56E-02 | -8.91±4.67 | 5.72E-02 |
| TG(14:1/14:1/20:2) | 1.17±0.52 | 2.58E-02 |  |  | -1.14±1.97 | 5.64E-01 |  |  |
| Cer(d18:1/20:1) | -1.58±0.72 | 2.80E-02 | -2.07±0.73 | 4.77E-03 | -5.28±2.93 | 7.28E-02 | -6.73±2.95 | 2.33E-02 |
| Cer(d18:0/24:0) | 0.95±0.47 | 4.36E-02 |  |  | -2.54±1.72 | 1.40E-01 |  |  |
| LPC(20:3/0:0) | 1.65±0.83 | 4.54E-02 |  |  | -7.42±3.37 | 2.83E-02 |  |  |
| LPE(0:0/24:1) | -1.54±0.77 | 4.60E-02 | -1.61±0.77 | 3.75E-02 | -1.82±3.18 | 5.68E-01 |  |  |
| LPC(20:0/0:0) | 1.71±0.87 | 4.93E-02 |  |  | -10.45±4.00 | 9.49E-03 | -9.07±4.29 | 3.55E-02 |
| LPS(16:0/0:0) | 1.91±0.98 | 5.21E-02 |  |  | -9.18±4.20 | 2.98E-02 | -7.28±4.45 | 1.03E-01 |
| LPE(0:0/18:3) | -1.28±0.67 | 5.74E-02 | -1.07±0.68 | 1.18E-01 | 1.33±2.93 | 6.50E-01 |  |  |
| LPE(0:0/18:2) | -1.50±0.79 | 5.82E-02 | -2.20±0.79 | 5.70E-03 | 2.16±3.26 | 5.07E-01 | 6.69±3.57 | 6.21E-02 |
| LPE(0:0/24:0) | 1.35±0.74 | 6.72E-02 |  |  | -3.36±2.92 | 2.51E-01 |  |  |
| CerP(d18:1/18:0) | 1.11±0.66 | 9.11E-02 |  |  | 0.08±2.40 | 9.73E-01 |  |  |
| LPC(18:3/0:0) | -1.15±0.73 | 1.13E-01 |  |  | -2.49±3.06 | 4.17E-01 |  |  |
| TG(14:1/14:1/22:3) | 0.81±0.57 | 1.53E-01 |  |  | -0.87±2.07 | 6.73E-01 |  |  |
| TG(14:1/14:1/22:2) | 0.98±0.70 | 1.61E-01 |  |  | -1.16±2.52 | 6.46E-01 |  |  |
| PC(20:2/22:6) | 1.20±0.95 | 2.05E-01 |  |  | -4.05±3.80 | 2.87E-01 |  |  |
| LPC(20:4/0:0) | 0.93±0.85 | 2.74E-01 |  |  | -6.61±3.58 | 6.56E-02 | -6.01±3.58 | 9.50E-02 |
| TG(14:0/18:0/22:4) | 0.61±0.58 | 2.92E-01 |  |  | -0.17±2.04 | 9.33E-01 |  |  |
| Cer(d18:1/16:1) | -0.93±0.95 | 3.29E-01 | -2.92±1.06 | 6.13E-03 | -1.41±3.86 | 7.15E-01 |  |  |
| Cer(d18:1/24:1) | -0.73±0.83 | 3.80E-01 | -1.59±0.85 | 6.16E-02 | -6.84±3.24 | 3.55E-02 | -6.44±3.24 | 4.77E-02 |
| PC(22:1/22:2) | -0.50±0.72 | 4.92E-01 | -1.17±0.73 | 1.09E-01 | -1.33±3.11 | 6.70E-01 |  |  |
| DG(14:1/20:0/0:0) | -0.45±0.83 | 5.87E-01 |  |  | -2.43±3.07 | 4.29E-01 |  |  |
| LPC(16:1/0:0) | 0.26±0.89 | 7.75E-01 |  |  | -3.44±3.92 | 3.80E-01 |  |  |
| DG(16:1/20:0/0:0) | -0.17±0.72 | 8.09E-01 |  |  | -2.24±2.66 | 4.00E-01 |  |  |
| LPC(18:2/0:0) | 0.21±0.90 | 8.15E-01 | -1.60±0.93 | 8.66E-02 | -5.71±3.68 | 1.22E-01 |  |  |
| DG(16:1/18:0/0:0) | -0.18±0.89 | 8.38E-01 |  |  | -3.99±3.34 | 2.32E-01 |  |  |
| DG(14:0/16:1/0:0) | 0.10±0.60 | 8.65E-01 |  |  | 2.55±2.74 | 3.53E-01 |  |  |
| HexCer(d18:1/22:0) | 0.00±0.48 | 9.94E-01 |  |  | 0.49±1.62 | 7.61E-01 |  |  |
| Estimates were calculated by applying a linear regression model. The empty cells were the variables excluded in multivariate regression based on backward stepwise. The lipid species refer to the metabolites associated with death or MACE in the internal training cohort. In adjusted analysis, sex, heart failure and hyperlipidemia statuses, glucose, LDLC, AST, BNP and HDLC levels, CCBs and β-blockers treatments were included as covariates to analyze the association of metabolites with LVEF; heart failure and hyperlipidemia statuses, glucose, β-blockers and ACEI treatments were used as covariates to analyze the association of metabolites with LVMI. | | | | | | | | |
